# Stable Gastric Pentadecapeptide BPC 157 Therapy for Primary Abdominal Compartment Syndrome in Rats

**DOI:** 10.3389/fphar.2021.718147

**Published:** 2021-12-13

**Authors:** Marijan Tepes, Slaven Gojkovic, Ivan Krezic, Helena Zizek, Hrvoje Vranes, Zrinko Madzar, Goran Santak, Lovorka Batelja, Marija Milavic, Suncana Sikiric, Ivica Kocman, Karol Simonji, Mariam Samara, Mario Knezevic, Ivan Barisic, Eva Lovric, Sanja Strbe, Antonio Kokot, Ivica Sjekavica, Toni Kolak, Anita Skrtic, Sven Seiwerth, Alenka Boban Blagaic, Predrag Sikiric

**Affiliations:** ^1^ Department of Surgery, General Hospital Nasice, Nasice, Croatia; ^2^ Department of Clinical Medicine, Faculty of Dental Medicine and Health Osijek, Osijek, Croatia; ^3^ PhD Program Translational Research in Biomedicine—TRIBE, School of Medicine, University of Split, Split, Croatia; ^4^ Department of Pharmacology, School of Medicine, University of Zagreb, Zagreb, Croatia; ^5^ Clinical Department of Surgery, Sestre Milosrdnice University Hospital Center, Zagreb, Croatia; ^6^ Department of Surgery, Faculty of Medicine, University of Osijek, Osijek, Croatia; ^7^ Department of Pathology, School of Medicine, University of Zagreb, Zagreb, Croatia; ^8^ Internal Diseases Clinic, Faculty of Veterinary Medicine Zagreb, Zagreb, Croatia; ^9^ Department of Anatomy and Neuroscience, Faculty of Medicine, J.J. Strossmayer University of Osijek, Osijek, Croatia; ^10^ Department of Diagnostic and Interventional Radiology, University Hospital Centre, Zagreb, Croatia; ^11^ Department of Surgery, School of Medicine, University of Zagreb, Zagreb, Croatia

**Keywords:** gastric pentadecapeptide BPC 157, primary abdominal compartment syndrome, rats, brain edema, lung edema

## Abstract

Recently, the stable gastric pentadecapeptide BPC 157 was shown to counteract major vessel occlusion syndromes, i.e., peripheral and/or central occlusion, while activating particular collateral pathways. We induced abdominal compartment syndrome (intra-abdominal pressure in thiopental-anesthetized rats at 25 mmHg (60 min), 30 mmHg (30 min), 40 mmHg (30 min), and 50 mmHg (15 min) and in esketamine-anesthetized rats (25 mmHg for 120 min)) as a model of multiple occlusion syndrome. By improving the function of the venous system with BPC 157, we reversed the chain of harmful events. Rats with intra-abdominal hypertension (grade III, grade IV) received BPC 157 (10 µg or 10 ng/kg sc) or saline (5 ml) after 10 min. BPC 157 administration recovered the azygos vein via the inferior–superior caval vein rescue pathway. Additionally, intracranial (superior sagittal sinus), portal, and caval hypertension and aortal hypotension were reduced, as were the grossly congested stomach and major hemorrhagic lesions, brain swelling, venous and arterial thrombosis, congested inferior caval and superior mesenteric veins, and collapsed azygos vein; thus, the failed collateral pathway was fully recovered. Severe ECG disturbances (i.e., severe bradycardia and ST-elevation until asystole) were also reversed. Microscopically, transmural hyperemia of the gastrointestinal tract, intestinal mucosa villi reduction, crypt reduction with focal denudation of superficial epithelia, and large bowel dilatation were all inhibited. In the liver, BPC 157 reduced congestion and severe sinusoid enlargement. In the lung, a normal presentation was observed, with no alveolar membrane focal thickening and no lung congestion or edema, and severe intra-alveolar hemorrhage was absent. Moreover, severe heart congestion, subendocardial infarction, renal hemorrhage, brain edema, hemorrhage, and neural damage were prevented. In conclusion, BPC 157 cured primary abdominal compartment syndrome.

## Introduction

We suggest that abdominal compartment syndrome (Depauw et al., 2019) is a multiple occlusion syndrome. Therefore, it is thought that by improving the function of the venous system with the stable gastric pentadecapeptide BPC 157 (Vukojevic et al., 2018; Gojkovic et al., 2020; Kolovrat et al., 2020; Gojkovic et al., 2021a; Knezevic et al., 2021a; Knezevic et al., 2021a; Gojkovic et al., 2021b; Knezevic et al., 2021b; Strbe et al., 2021), the chain of harmful events in abdominal compartment syndrome can be reversed.

The stable gastric pentadecapeptide BPC 157 was chosen and tested in this study due to its beneficial effects in major vessel occlusion syndromes (Vukojevic et al., 2018; Gojkovic et al., 2020; Kolovrat et al., 2020; Gojkovic et al., 2021a; Knezevic et al., 2021a; Knezevic et al., 2021a; Gojkovic et al., 2021b; Knezevic et al., 2021b; Strbe et al., 2021) and as a prototypic cytoprotective peptide (for review, see Sikiric et al., 1993a; Sikiric et al., 2006; Sikiric et al., 2010; Sikiric et al., 2011; Sikiric et al., 2012; Sikiric et al., 2013; Sikiric et al., 2014; Sikiric et al., 2016; Sikiric et al., 2017; Sikiric et al., 2018; Sikiric et al., 2020a; Sikiric et al., 2020b; Seiwerth et al., 2014; Seiwerth et al., 2018; Seiwerth et al., 2021; Kang et al., 2018; Park et al., 2020; Gwyer et al., 2019, Vukojevic et al., 2022).

To fully model intra-abdominal hypertension syndrome (continuous intraperitoneal insufflation of ordinary air), these occlusion syndromes have been induced peripherally (Vukojevic et al., 2018; Gojkovic et al., 2020; Kolovrat et al., 2020; Knezevic et al., 2021a; Knezevic et al., 2021a; Knezevic et al., 2021b) or centrally (Gojkovic et al., 2021a) and both peripherally and centrally (Gojkovic et al., 2021b; Strbe et al., 2021). Specific occlusion syndrome induction can be performed by the occlusion of a major vein (Vukojevic et al., 2018; Gojkovic et al., 2020; Gojkovic et al., 2021a; Knezevic et al., 2021b) or an artery (Knezevic et al., 2021a), or with both artery and vein occlusion (Kolovrat et al., 2020; Knezevic et al., 2021a), or by intragastric application of absolute alcohol (Gojkovic et al., 2021b) and intraperitoneal application of lithium overdose (Strbe et al., 2021).

Considering the effects of BPC 157 therapy peripherally and centrally (Vukojevic et al., 2018; Gojkovic et al., 2020; Kolovrat et al., 2020; Gojkovic et al., 2021a; Knezevic et al., 2021a; Knezevic et al., 2021a; Gojkovic et al., 2021b; Knezevic et al., 2021b; Strbe et al., 2021), in rats with severely increased intra-abdominal pressure, i.e., primary abdominal compartment syndrome, we attempted to introduce a therapy for compressed essential vessel tributaries, both arterial and venous (peripherally and centrally), due to occluded major veins and arteries, in order to prevent the consequent noxious syndrome, both peripherally and centrally. Otherwise, intra-abdominal hypertension adversely affects many organs, such as the brain, heart, lungs, kidneys, and gastrointestinal tract (Cullen et al., 1989), progressing to lethal levels. As abdominal compartment syndrome leads to organ failure at an intra-abdominal pressure of 20 mmHg (Hunter and Damani, 2004; Hedenstierna and Larsson, 2012), to assess the degree of severity that can be treated with this therapy, higher intra-abdominal pressures of 25, 30, 40, and 50 mmHg were also used. It was found that systemic and splanchnic blood flow and afferent hepatic flow were reduced as the intra-abdominal pressure rose; i.e., liver blood flow decreased by 39% when pneumoperitoneum increased from 10 to 15 mmHg and liver ischemic injury occurred (Chen et al., 2017).

Furthermore, as an immediate effect, the abdominal, thoracic, and cranial cavities interact with each other (Depauw et al., 2019), and increased intra-abdominal pressure causes an increase in intracranial pressure (Malbrain and Wilmer, 2007; Scalea et al., 2007; Youssef et al., 2012; Chen et al., 2020). Increased intra-abdominal pressure also increases intrathoracic pressure, which is rapidly transmitted up through the venous system, thereby further increasing intracranial pressure (Malbrain and Wilmer, 2007; Scalea et al., 2007; Youssef et al., 2012; Chen et al., 2020). Thus, although not specifically indicated, these findings support the rapid improvement of venous system function as an essential common point to prevent and reverse the noxious chain of events and attenuate all harmful consequences.

Thus, it may be that maintained increased intra-abdominal pressure causes widespread dysfunction, which would be similar to the severe syndromes observed in rats with the occlusion of peripheral vessels (Vukojevic et al., 2018; Gojkovic et al., 2020; Kolovrat et al., 2020; Knezevic et al., 2021a; Knezevic et al., 2021a; Knezevic et al., 2021b) and central vessels (Gojkovic et al., 2021a) or after the intragastric application of absolute alcohol (Gojkovic et al., 2021b) and intraperitoneal application of the lithium overdose (Strbe et al., 2021). These peripheral and central deficits can include severe gastrointestinal lesions, intracranial (superior sagittal sinus) hypertension, brain swelling and lesions, portal and caval hypertension, aortic hypotension, peripheral and central thrombosis, inferior caval vein and superior mesenteric vein congestion, azygos vein failure, electrocardiogram (ECG) disturbances, and heart, lung, liver, and kidney lesions (Vukojevic et al., 2018; Gojkovic et al., 2020; Kolovrat et al., 2020; Gojkovic et al., 2021a; Knezevic et al., 2021a; Knezevic et al., 2021a; Gojkovic et al., 2021b; Knezevic et al., 2021b; Strbe et al., 2021). Syndrome development and treatment with BPC 157 have been demonstrated in a variety of procedures inducing vessel occlusion (Vukojevic et al., 2018; Gojkovic et al., 2020; Kolovrat et al., 2020; Gojkovicc et al., 2021a; Knezevic et al., 2021a; Knezevic et al., 2021a; Gojkovic et al., 2021b; Knezevic et al., 2021b; Strbe et al., 2021). These syndromes, specifically those induced by vessel occlusion and subsequently treated with BPC 157, include inferior caval vein syndrome (Vukojevic et al., 2018), Pringle maneuver ischemia, reperfusion (Kolovrat et al., 2020), Budd–Chiari syndrome (Gojkovic et al., 2020), superior sagittal sinus occlusion (Gojkovic et al., 2021a), and superior mesenteric artery and/or vein occlusion (Knezevic et al., 2021a; Knezevic et al., 2021a; Knezevic et al., 2021b). The bypassing loops appear to be reliant on the corresponding injurious occlusion and reestablish blood flow to compensate for vessel occlusion and to reduce syndrome severity (Vukojevic et al., 2018; Gojkovic et al., 2020; Kolovrat et al., 2020; Gojkovic et al., 2021a; Knezevic et al., 2021a; Knezevic et al., 2021a; Gojkovic et al., 2021b; Knezevic et al., 2021b; Strbe et al., 2021). Previously, we showed this for the left ovarian vein (i.e., inferior caval vein syndrome (Vukojevic et al., 2018)), the inferior mesenteric vein in the portocaval shunt (Kolovrat et al., 2020), and the azygos vein in the superior-inferior caval vein shunt (Gojkovic et al., 2020; Gojkovic et al., 2021a; Gojkovic et al., 2021b; Strbe et al., 2021). More specifically, the superior mesenteric vein, inferior and superior anterior pancreaticoduodenal, pyloric vein, and portal vein in the superior mesenteric vein-portal vein shunt reestablish the interrupted superior mesenteric and portal vein pathway (the occluded end of the superior mesenteric vein) (Knezevic et al., 2021b). The inferior mesenteric artery and inferior anterior pancreaticoduodenal artery are alternative pathways in the case of an occluded superior mesenteric artery (Knezevic et al., 2021a). With simultaneous occlusion of both superior mesenteric vessels, i.e., the artery and the vein, both pathways, arterial and venous, are activated (Knezevic et al., 2021a). Centrally (para)sagittal venous collateral circulation appears with an occluded superior sagittal sinus (Gojkovic et al., 2021a). It has been theorized that BPC 157 therapy could likely represent a “bypassing key,” by rapidly activating bypassing pathways and abrogating the complex syndrome induced by simultaneous occlusion of essential arterial and venous tributaries. Likewise, it has been theorized that this “bypassing key” appears to be an effect of the essential endothelial protective capacity of BPC 157. BPC 157, as a novel and relevant cytoprotective mediator, rapidly activates collateral bypassing pathways and alleviates vessel occlusion syndromes (Vukojevic et al., 2018; Gojkovic et al., 2020; Kolovrat et al., 2020; Gojkovic et al., 2021a; Knezevic et al., 2021a; Knezevic et al., 2021a; Gojkovic et al., 2021b; Knezevic et al., 2021b; Strbe et al., 2021). As such, with BPC 157 therapy, endothelial protection (as a shared effect of cytoprotective agents (Robert, 1979; Szabo et al., 1985)) and the cytoprotection theory maxim “endothelium maintenance → epithelium maintenance” (Robert, 1979; Szabo et al., 1985) may have additional significance. Namely, Robert’s and Szabo’s original maxim (“endothelium maintenance → epithelium maintenance”) may be further promoted. Therefore, we reported evidence about blood vessel recruitment and activation (“running”) toward the site of injury, also described as bypassing occlusion via alternative pathways (Vukojevic et al., 2018; Gojkovic et al., 2020; Kolovrat et al., 2020; Gojkovic et al., 2021a; Knezevic et al., 2021a; Knezevic et al., 2021a; Gojkovic et al., 2021b; Knezevic et al., 2021b; Strbe et al., 2021). Consequently, compensatory activated collateral blood vessels and reorganized blood flow following BPC 157 treatment in rats with the occluded major peripheral vessel(s) or central vessels reduced superior sagittal sinus, portal and caval hypertension, aortal hypotension, progressive venous and arterial thrombosis peripherally and centrally, and ECG disturbances. Markedly, multiple organ lesions in the heart, lung, liver, kidney, and gastrointestinal tract, in particular, as well as brain lesions, were attenuated, and oxidative stress was reduced in tissues (Vukojevic et al., 2018; Gojkovic et al., 2020; Kolovrat et al., 2020; Gojkovic et al., 2021a; Knezevic et al., 2021a; Knezevic et al., 2021a; Knezevic et al., 2021b; Strbe et al., 2021).

The many blood vessels identified as being activated by specific pathways following a given vessel injury require a regularly applicable therapy, with beneficial effects dependent on, but not limited to, occlusion of a particular vessel (Sikiric et al., 2018). With BPC 157 therapy, this point was envisaged by the consistent reduction of the whole “occlusive-like” syndrome that regularly follows the intragastric application of absolute alcohol in rats (Gojkovic et al., 2021b) and intraperitoneal application of the lithium overdose (Strbe et al., 2021). Consequently, we observed that this beneficial effect, after direct injury (permanent ligation) applied to one or two major vessels, could instantly oppose more general damage (maintained intra-abdominal hypertension, either high (grade III) or very high (grade IV)), as all blood vessels which can be compressed with increased intra-abdominal pressure. Therefore, a “bypassing key,” i.e., an activated azygos vein as a rescuing pathway, avoiding both the lung and liver and also noted in Budd–Chiari syndrome (i.e., suprahepatic occlusion of the inferior caval vein) (Gojkovic et al., 2020), combines the inferior caval vein and superior caval vein via direct blood delivery. Thus, activated azygos vein shunt could reorganize blood flow and instantly attenuate the consequences of maintained high intra-abdominal pressure, both peripherally and centrally.

BPC 157s endothelial effects and its function as a “bypassing key” (Sikiric et al., 2018) are strongly supported by its interaction with the nitric oxide (NO) system (for a review, see Sikiric et al., 2014). The most recent demonstration of the impact of BPC 157 on vasomotor tone was carried out through BPC 157-specific activation of the Src-caveolin-1-endothelial NO synthase (eNOS) pathway (Hsieh et al., 2020). BPC 157 acts as a membrane stabilizer and free radical scavenger and reduces leaky gut syndrome, as shown in gastrointestinal tract cytoprotective studies (Park et al., 2020). BPC 157 also has a curative effect due to interactions with several molecular pathways (Tkalcević et al., 2007; Chang et al., 2011, 2014; Huang et al., 2015; Hsieh et al., 2017; Kang et al., 2018; Vukojevic et al., 2018; Wang et al., 2019; Cesarec et al., 2013; Hsieh et al., 2020; Park et al., 2020; Vukojevic et al., 2020; Wu et al., 2020).

Thus, we assessed BPC 157 therapy as a curative principle in rats with established permanent intra-abdominal hypertension. As confirmation, we used the crisis that occurred with the high intra-abdominal pressure-induced syndrome, in which intra-abdominal hypertension simultaneously affected all abdominal vessels and organs for a considerable period and restrained the ability to recruit alternative pathways, such that a deadly situation was created before therapy initiation.

## Materials and Methods

### Animals

This study was conducted with 12-week-old, 200 g body weight, male Albino Wistar rats, randomly assigned at six rats/group/interval. Rats were bred in-house at the Animal Pharmacology Facility, School of Medicine, Zagreb, Croatia. The animal facility is registered with the Veterinary Directorate (Reg. No: HR-POK-007). Laboratory rats were acclimated for five days and randomly assigned to their respective treatment groups. Laboratory animals were housed in polycarbonate (PC) cages under conventional laboratory conditions at 20–24°C, relative humidity of 40–70%, and noise level of 60 dB. Each cage was identified with dates, number of studies, group, dose, number, and sex of each animal. Fluorescent lighting provided illumination 12 h per day. Standard good laboratory practice (GLP) diet and fresh water were provided *ad libitum*. Animal care was in compliance with standard operating procedures (SOPs) of the Animal Pharmacology Facility and the European Convention for the Protection of Vertebrate Animals used for Experimental and Other Scientific Purposes (ETS 123).

This study was approved by the local ethics committee. Ethical principles of the study complied with the European Directive 010/63/E, the Law on Amendments to the Animal Protection Act (Official Gazette 37/13), the Animal Protection Act (Official Gazette 135/06), the ordinance on the protection of animals used for scientific purposes (Official Gazette 55/13), Federation of European Laboratory Animal Science Associations (FELASA) recommendations, and the recommendations of the Ethics Committee of the School of Medicine, University of Zagreb. The experiments were assessed by observers blinded as to the treatment.

### Drugs

Medication was administered as described previously (Vukojevic et al., 2018; Gojkovic et al., 2020; Kolovrat et al., 2020; Gojkovic et al., 2021a; Knezevic et al., 2021a; Knezevic et al., 2021a; Gojkovic et al., 2021b; Knezevic et al., 2021b), without the use of a carrier or peptidase inhibitor, for stable gastric pentadecapeptide BPC 157 (10 µg or 10 ng/kg subcutaneously), a partial sequence of the human gastric juice protein BPC, which is freely soluble in water at pH 7.0 and in saline. BPC 157 (GEPPPGKPADDAGLV, molecular weight 1,419; Diagen, Slovenia) was prepared as a peptide with 99% high-performance liquid chromatography (HPLC) purity, with 1-des-Gly peptide being the main impurity. The dose and application regimens were as described previously (Duzel et al., 2017; Amic et al., 2018; Drmic et al., 2018; Vukojevic et al., 2018; Sever et al., 2019; Cesar et al., 2020; Gojkovic et al., 2020; Kolovrat et al., 2020; Vukojevic et al., 2020).

### Experimental Protocol

In deeply anesthetized rats (intraperitoneal (ip) injected 40 mg/kg thiopental (Rotexmedica, Germany) and 10 mg/kg diazepam (Apaurin; Krka, Slovenia)), we induced abdominal compartment syndrome by intraperitoneal insufflation of ordinary air controlled by a manual and digital manometer with a data logger connected to a computer (DD890, Dostmann Electronic GmbH, Germany) and maintained high abdominal pressure at 25 mmHg for 120 min before sacrifice, with a pressure measurement interval of 1 s. High abdominal pressure at 25, 30, 40, or 50 mmHg was maintained until sacrifice at 60 min (25 mmHg), 30 min (30 mmHg, 40 mmHg), or 15 min (50 mmHg). Rats received BPC 157 (10 µg or 10 ng/kg subcutaneously) or saline (5 ml) at 10 min abdominal compartment syndrome-time. Alternatively, using esketamine anesthesia (40 mg/kg esketamine (Rotexmedica, Germany) and 10 mg/kg diazepam (Apaurin; Krka, Slovenia) intraperitoneally), we induced abdominal compartment syndrome as described before and maintained high abdominal pressure at 25 mmHg for 120 min before sacrifice. Medication (BPC 157 (10 µg or 10 ng/kg sc) or saline (5 ml)) was given after 10 min of high abdominal pressure.

Recordings of brain swelling were performed in rats before sacrifice after complete calvariectomy was performed (Gojkovic et al., 2021a; Knezevic et al., 2021a; Knezevic et al., 2021a; Knezevic et al., 2021b). Briefly, six burr holes were drilled in three horizontal lines, all of them medially to the superior temporal lines and temporalis muscle attachments. The two rostral burr holes were placed just basal from the posterior interocular line, the two basal burr holes were placed just rostral to the lambdoid suture (and transverse sinuses) on both sides, respectively, and the two middle burr holes were placed in line between the basal and rostral burr holes.

Rats were laparatomized before sacrifice for the corresponding presentation of the peripheral vessels (azygos vein, superior mesenteric vein, portal vein, inferior caval vein, and abdominal aorta). The recording was performed with a camera attached to a VMS-004 Discovery Deluxe USB microscope (Veho, United States) at the end of the experiment and assessed as before (Gojkovic et al., 2021a; Knezevic et al., 2021a; Knezevic et al., 2021a; Knezevic et al., 2021b; Strbe et al., 2021).

### Superior Sagittal Sinus, Portal, Superior Mesenteric, and Caval Vein, and Abdominal Aorta Pressure Recording

As described before (Vukojevic et al., 2018; Gojkovic et al., 2020; Kolovrat et al., 2020; Gojkovic et al., 2021a; Knezevic et al., 2021a; Knezevic et al., 2021a; Gojkovic et al., 2021b; Knezevic et al., 2021b; Strbe et al., 2021), recordings were made in deeply anesthetized rats with a cannula (BD Neoflon™ Cannula) connected to a pressure transducer (78534C MONITOR/TERMINAL; Hewlett Packard, United States), inserted into the portal vein, inferior caval vein, and superior sagittal sinus, as well as the abdominal aorta at the level of the bifurcation at 15, 30, 60, or 120 min ACS-time. For superior sagittal sinus pressure recording, we made a single burr hole in the rostral part of the sagittal suture, above the superior sagittal sinus, and cannulated the superior sagittal sinus anterior part using a Braun intravenous cannula; then, we laparatomized the rat for portal vein, inferior vena cava, and abdominal aorta pressure recording.

Notably, normal rats exhibited a superior sagittal sinus pressure of −24 to −27 mmHg and superior mesenteric pressure and portal pressure of 3–5 mmHg similar to that of the inferior vena cava, though with values at least 1 mmHg higher in the portal vein. By contrast, abdominal aorta blood pressure values were 100–120 mm Hg at the level of the bifurcation (Vukojevic et al., 2018; Gojkovic et al., 2020; Kolovrat et al., 2020; Gojkovic et al., 2021a; Knezevic et al., 2021a; Knezevic et al., 2021a; Gojkovic et al., 2021b; Knezevic et al., 2021b; Strbe et al., 2021).

### ECG Recording

ECGs were recorded continuously in deeply anesthetized rats for all three main leads, by positioning stainless steel electrodes on all four limbs using an ECG monitor with a 2090 programmer (Medtronic, United States) connected to a Waverunner LT342 digital oscilloscope (LeCroy, United States) at 30 min ligation time. This arrangement enabled precise recordings, measurements, and analysis of ECG parameters (Vukojevic et al., 2018; Gojkovic et al., 2020; Kolovrat et al., 2020; Gojkovic et al., 2021a; Knezevic et al., 2021a; Knezevic et al., 2021a; Gojkovic et al., 2021b; Knezevic et al., 2021b; Strbe et al., 2021). The time until extreme bradycardia and asystole was assessed.

### Thrombus Assessment

Following sacrifice, the superior sagittal sinus and peripherally the portal vein, external jugular vein, inferior caval vein, superior mesenteric vein, hepatic vein, superior mesenteric artery, hepatic artery, and abdominal aorta were removed from the rats, and the clots were weighed (Vukojevic et al., 2018; Gojkovic et al., 2020; Kolovrat et al., 2020; Gojkovic et al., 2021a; Knezevic et al., 2021a; Knezevic et al., 2021a; Gojkovic et al., 2021b; Knezevic et al., 2021b; Strbe et al., 2021).

### Brain Volume and Vessel Presentation

Brain volume and vessel presentation were proportional to the change in the brain or vessel surface area. The presentation of the brain and peripheral vessels (superior mesenteric vein, portal vein, inferior caval vein, azygos vein, and abdominal aorta) was recorded in deeply anesthetized rats, with a camera attached to a VMS-004 Discovery Deluxe USB microscope (Veho, United States) (Gojkovic et al., 2021a; Knezevic et al., 2021a; Knezevic et al., 2021a; Gojkovic et al., 2021b; Knezevic et al., 2021b; Strbe et al., 2021). The border of the brain in the image was marked using ImageJ software and then the surface area of the brain was measured. This was done with brain images for both the control (saline) group and treated (BPC 157) group of rats at same intervals after the application and at the time of sacrifice. The arithmetic mean of the surface areas was calculated for both groups. Then, the ratio of these two areas was calculated as ( $\frac{A_{con}}{A_{bpc}}$ ), where A_con_ is the arithmetic mean brain area of the control group and A_bpc_ is the arithmetic mean brain area of the treated group. Starting from the square-cube law equations *[1] [2]*, an equation for the change in brain volume proportional to the change in brain surface area *[6]* was derived. In expressions [*1–5*], *l* is defined as any arbitrary one-dimensional length of the brain (for example, rostrocaudal length of the brain), used only for defining the one-dimensional proportion (l_2_/l_1_) between two observed brains and as an inter-factor (and because of that not measured *[6]*) for deriving final expression *[6]*. The procedure was as follows: ${\text{ A}}_{2}={\text{A}}_{1}\times {\left(\frac{{\text{l}}_{2}}{{\text{l}}_{1}}\right)}^{2}$ *[1]* (square-cube law), ${\text{V}}_{2}={\text{V}}_{1}\times {\left(\frac{{\text{l}}_{2}}{{\text{l}}_{1}}\right)}^{3}$ *[2]* (square-cube law), $\frac{{\text{A}}_{2}}{{\text{A}}_{1}}={\left(\frac{{\text{l}}_{2}}{{\text{l}}_{1}}\right)}^{2}$ [3] (from [1], after dividing both sides by A_1_), $\frac{{\text{l}}_{2}}{{\text{l}}_{1}}=\sqrt{\frac{{\text{A}}_{2}}{{\text{A}}_{1}}}$ *[4]* (from *[3]*, after taking the square root of both sides), $\frac{{\text{V}}_{2}}{{\text{V}}_{1}}={\left(\frac{{\text{l}}_{2}}{{\text{l}}_{1}}\right)}^{3}$ *[5]* (from *[2]*, after dividing both sides by V_1_), and $\frac{{\text{V}}_{2}}{{\text{V}}_{1}}={\left(\sqrt{\frac{{\text{A}}_{2}}{{\text{A}}_{1}}}\;\right)}^{3}$ *[6]* (after incorporating expression *[4]* into equation *[5]*).

### Gross Assessment of Gastrointestinal Lesions

A camera attached to a VMS-004 Discovery Deluxe USB microscope (Veho, United States) was used for recording. In deeply anesthetized rats, laparatomized before sacrifice, we assessed the gross lesions in the gastrointestinal tract and in the stomach (sum of the longest diameters, mm) (Gojkovic et al., 2020; Kolovrat et al., 2020; Gojkovic et al., 2021a; Knezevic et al., 2021a; Knezevic et al., 2021a; Gojkovic et al., 2021b; Knezevic et al., 2021b; Strbe et al., 2021).

### Liver and Spleen Weights

Liver and spleen weights are expressed as a percentage of total body weight (for normal rats, liver, 3.2–4.0%; spleen, 0.20–0.26%).

## Microscopy

From rats, at end of the experiment, the brain, liver, kidney, stomach, duodenum, jejunum, colon, rectum, lungs, and heart were fixed in 10% neutral buffered formalin (pH 7.4) at room temperature for 24 h. Representative tissue specimens were embedded in paraffin, sectioned at 4 μm, stained with hematoxylin and eosin (H&E), and evaluated by light microscopy using an Olympus 71 digital camera and an Olympus BX51 microscope (Japan) acquiring digital images saved as uncompressed 24-bit RGB TIFF files.

### Analysis of Central Nervous System Karyopyknotic Cells

Modified Bielschowsky’s silver staining and Klüver–Barrera staining (using Klüver–Barrera Luxol fast blue) were performed to demonstrate argentophilic neurites, axonal spheroids, and neuronal cell bodies, particularly in brain karyopyknotic areas (https://journals.sagepub.com/doi/pdf/10.1038/jcbfm.1995.128) (file:///F:/ACS%20manuscript/CVI_rat_phd_nedergaard1987.pdf).

The brain was dissected according to NTP-7 at Levels 3 and 6 with neuroanatomic subsites presented in certain brain sections using coronal sections with three mandatory sections (Eustis et al., 2017; Gojkovic et al., 2021a; Knezevic et al., 2021a; Knezevic et al., 2021a; Gojkovic et al., 2021b; Knezevic et al., 2021b; Strbe et al., 2021) and analyzed using a semiquantitative neuropathological scoring system, as previously described (Bona et al., 1998; Gojkovic et al., 2021a; Knezevic et al., 2021a; Knezevic et al., 2021a; Gojkovic et al., 2021b; Knezevic et al., 2021b; Strbe et al., 2021), the and combined score (0–8) = the sum of the analyzed affected areas (0–4) and karyopyknotic cells in the brain areas (0–4), as follows. Specifically, analyzed were the affected brain areas (0–4), cerebral (NTP-7, Level 3), cerebellar cortex (NTP-7, Level 6), and hippocampus, thalamus, and hypothalamus (NTP-7, Level 3) as follows (score 0 indicates no histopathologic change): score 1: small, patchy, complete, or incomplete infarcts (≤10% of the area affected); score 2: partly confluent or incomplete infarcts (20–30% of the area affected); score 3: large confluent complete infarcts (40–60% of the area affected); score 4: in cortex total disintegration of the tissue and the hypothalamus, thalamus, and hippocampus large complete infarcts (˃75% of the area affected). Analyzed were karyopyknotic cells in the affected brain areas (0–4), cerebral (NTP-7, Level 3), cerebellar cortex (NTP-7, Level 6), and hippocampus, thalamus, and hypothalamus (NTP-7, Level 3) as follows (score 0 indicates no change): score 1: a few karyopyknotic of neuronal cells (≤20%); score 2: patchy areas of karyopyknotic cells (50%); score 3: more extensive karyopyknotic areas (75%); score 4: complete infarction (100%).

The neuronal pathological changes were also observed in the acquired digital images saved as uncompressed 24-bit RGB TIFF files in the software program AnalySIS (Olympus Soft Imaging System GmbH, Münster, Germany) performing quantitative analysis of neuronal damage in the karyopyknotic areas. The neurons of the cortical cerebral, cerebellar region, hippocampus, and hypothalamus were counted in 10 different high-powered fields (HPF, 400x) and 3 to 5 serial sections of each sample were used to do the count as described in https://www.ncbi.nlm.nih.gov/pmc/articles/PMC5303860/. The field size was 0.24 μm^2^.

Lung histology. A scoring system was used to grade the degree of lung injury in lung tissue analysis (Gojkovic et al., 2021a; Knezevic et al., 2021a; Knezevic et al., 2021a; Gojkovic et al., 2021b; Knezevic et al., 2021b). Features included focal thickening of the alveolar membranes, congestion, pulmonary edema, intra-alveolar hemorrhage, interstitial neutrophil infiltration, and intra-alveolar neutrophil infiltration. Each feature was assigned a score from 0 to 3 based on its absence (0) or presence to a mild (1), moderate (2), or severe (3) degree, and a final histology score was determined (Murao et al., 2003).

Renal, liver, and heart histology. The criteria renal injury was based on the degeneration of Bowman’s space and glomeruli, degeneration of the proximal and distal tubules, vascular congestion, and interstitial edema (Gojkovic et al., 2021a; Knezevic et al., 2021a; Knezevic et al., 2021a; Gojkovic et al., 2021b; Knezevic et al., 2021b; Strbe et al., 2021). The criteria for liver injury were vacuolization of hepatocytes and pyknotic hepatocyte nuclei, activation of Kupffer cells, and enlargement of sinusoids. Each specimen was scored using a scale ranging 0–3 (0: none; 1: mild; 2: moderate; 3: severe) for each criterion, and a final histology score was determined (Ibrahim, et al., 2010; Gojkovic et al., 2021a; Gojkovic et al., 2021b; Knezevic et al., 2021a; Knezevic et al., 2021b; Knezevic et al., 2021a; Strbe et al., 2021). Cardiac lesion estimation was based on the dilatation and congestion of blood vessels within the myocardium and coronary arteries using a scale ranging 0–3 (0: none; 1: mild; 2: moderate; 3: severe) (Gojkovic et al., 2021a; Knezevic et al., 2021a; Knezevic et al., 2021a; Gojkovic et al., 2021b; Knezevic et al., 2021b; Strbe et al., 2021).

Gastrointestinal histology. As previously described (Gojkovic et al., 2020; Kolovrat et al., 2020; Gojkovic et al., 2021a; Knezevic et al., 2021a; Knezevic et al., 2021a; Gojkovic et al., 2021b; Knezevic et al., 2021b; Strbe et al., 2021), intestinal tissue damage was analyzed using a histologic scoring scale adapted from Chui and coworkers (Chui et al., 1970) on a scale of 0–5 (normal to severe) in three categories (mucosal injury, inflammation, and hyperemia/hemorrhage) for a total score of 0–15, as described by Lane and coworkers (Lane et al., 1997). Morphologic features of mucosal injury were based on different grades of epithelial lifting, villi denudation, and necrosis; grades of inflammation were graded from focal to diffuse according to lamina propria infiltration or subendothelial infiltration; hyperemia/hemorrhage was graded from focal to diffuse according to lamina propria or subendothelial localization. In addition, the villi height was assessed as well (normal villi height as indicated before (Sever et al., 2009; Teshfam et al., 2010)).

### Statistical Analysis

Statistical analysis was performed by parametric one-way analysis of variance (ANOVA), with the Newman–Keuls *post hoc* test or the non-parametric Kruskal–Wallis test and subsequently the Mann–Whitney *U* test to compare groups. Values are presented as the mean ± standard deviation (SD) and as the minimum/median/maximum. To compare the frequency difference between groups, the chi-squared test or Fischer’s exact test was used. *p* < 0.05 was considered statistically significant.

## Results

We revealed that, despite permanently increased intra-abdominal hypertension (grade III and grade IV), a perilous syndrome occurred peripherally and centrally, the reversal of the abdominal compartment syndrome induced by the stable gastric pentadecapeptide BPC 157 application was quite consistent. With sustained increased intra-abdominal pressures and pentadecapeptide BPC 157 application, otherwise imminent abdominal compartment syndrome (i.e., 25 mmHg or 30 mmHg, or 40 mmHg or 50 mmHg for 25, 30, and 60 min (thiopental) and for 120 min (esketamine)) did not appear. This was seen with the portal, caval, aortal, and superior sagittal sinus pressure assessment, reduced major ECG disturbances, nearly abrogated arterial and vein thrombosis, and preserved presentation of the brain, heart, lungs, liver, kidneys, and gastrointestinal tract, with no lethal outcomes despite the permanent maintenance of high intra-abdominal pressure. Both BPC 157 regimens (µg and ng) provided a similar therapeutic effect in all of the investigated protocols of abdominal compartment syndrome.

Commonly, all increased intra-abdominal pressures (i.e., 25, 30, 40, and 50 mmHg) produced a highly noxious syndrome, which occurred both peripherally and centrally. This noxious syndrome resembled the major vessel occlusion-induced syndromes (Vukojevic et al., 2018; Gojkovic et al., 2020; Kolovrat et al., 2020; Gojkovic et al., 2021a; Knezevic et al., 2021a; Knezevic et al., 2021a; Knezevic et al., 2021b) or “occlusion-like” syndromes that appear after intragastric application of absolute alcohol (Gojkovic et al., 2021b) and intraperitoneal application of lithium overdose (Strbe et al., 2021), in particular, similar to the acute Budd–Chiari syndrome and acute suprahepatic inferior caval vein occlusion (Gojkovic et al., 2020). Contrarily, in rats with high intra-abdominal pressure, the application of BPC 157 had a considerable therapeutic effect. For this effect, in all BPC 157-treated rats, the common key finding may be the rapidly activated azygos vein collateral pathway, which combined the inferior caval vein and left superior caval vein, to reverse the rapid presentation of this deadly syndrome.

### Blood Pressure Disturbances

**FIGURE 1:**
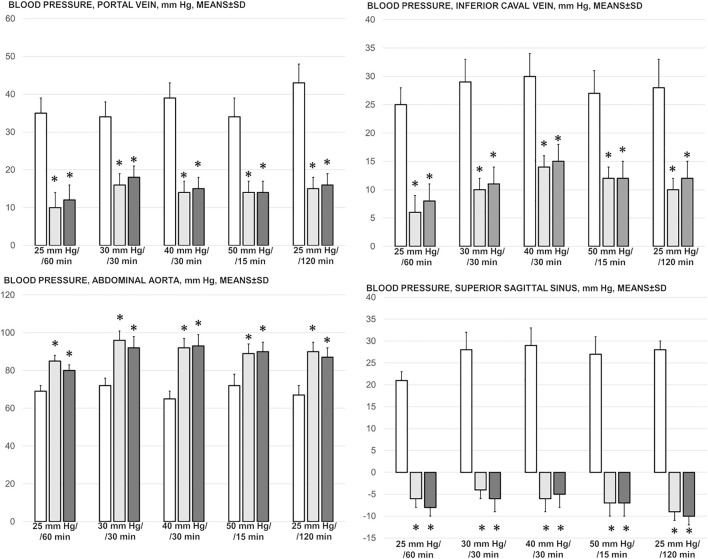
Blood pressure, mmHg (in the superior sagittal sinus (SSS), portal vein (PV), abdominal aorta (AA), inferior caval vein (ICV)), in the thiopental-anesthetized rats with the increased intra-abdominal pressures at 50 mmHg for 25 min, at 30 mmHg or 40 mmHg for 30 min, and at 25 mmHg for 60 min increased intra-abdominal pressures-time, and in the esketamine-anesthetized rats with the increased intra-abdominal pressures at the 25 mmHg for 120 min increased intra-abdominal pressures-time, following medication (BPC 157 10 μg/kg (light gray bars), 10 ng/kg (dark gray bars); saline 5 ml/kg (white bars)) given subcutaneously at 10 min increased intra-abdominal pressures-time. Means ± SD, *P˂0.05, vs. control.

Perceived as a cause-consequence relation, the important evidence is that BPC 157 reduced blood pressure disturbances that were induced by increased intra-abdominal pressures, shown to be quite severe and noted peripherally (portal and caval hypertension, aortal hypotension) as well centrally (superior sagittal sinus hypertension) (Figure 1). The severely increased pressure values in the portal vein, inferior caval vein, and superior sagittal sinus, as well as the decreased pressure values in the abdominal aorta, were markedly attenuated with BPC 157 application.

### Collateral Pathways, Blood Vessels, and Brain Gross Presentation

**FIGURE 2:**
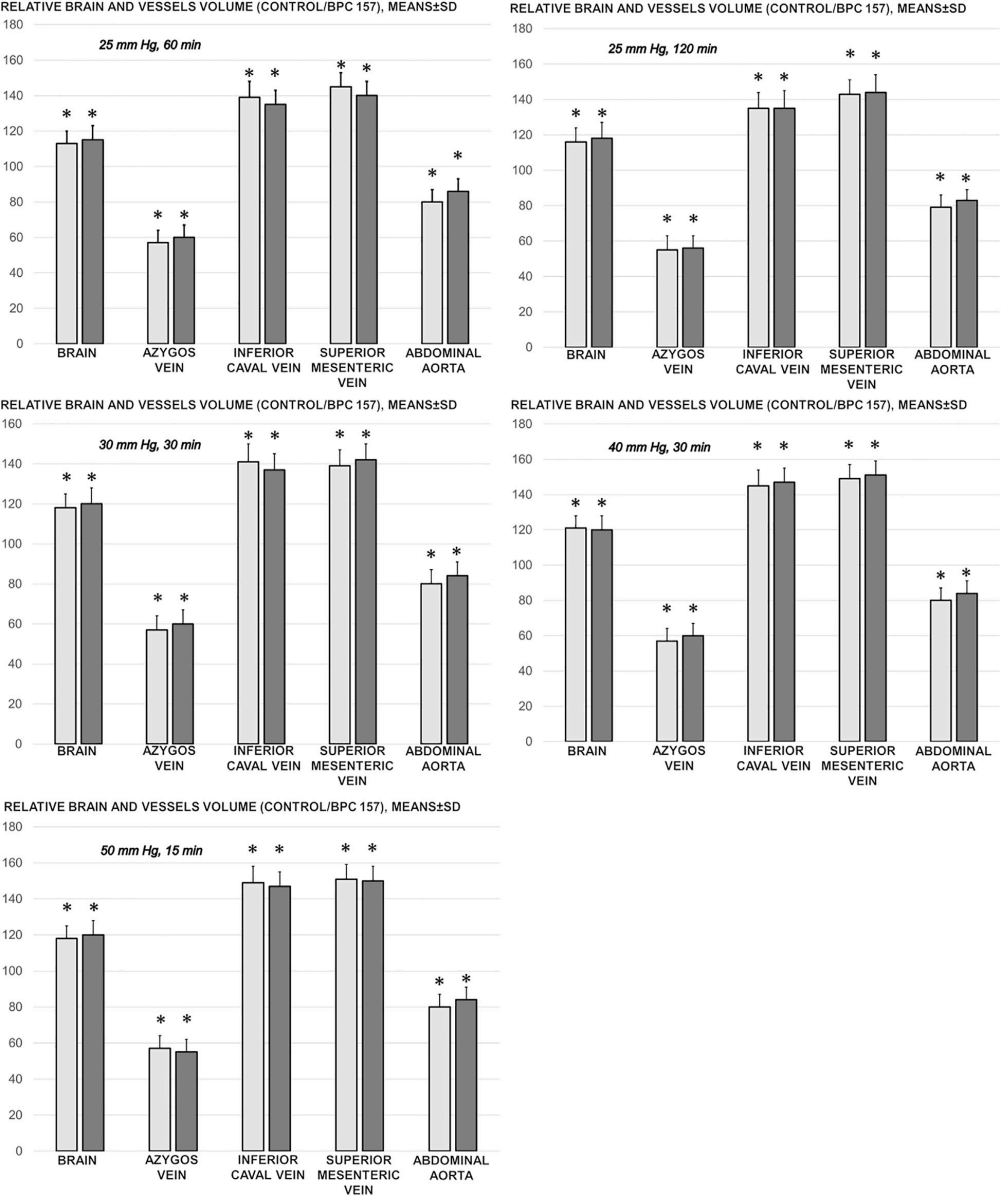
Relative brain and vessels volume (volume control/volume BPC 157, %) in the thiopental-anesthetized rats with the increased intra-abdominal pressures at 50 mmHg for 25 min, at 30 mmHg or 40 mmHg for 30 min, and at 25 mmHg for 60 min increased intra-abdominal pressures-time, and in the esketamine-anesthetized rats with the increased intra-abdominal pressures at 25 mmHg for 120 min increased intra-abdominal pressures-time, following medication (BPC 157 10 μg/kg (light gray bars), 10 ng/kg (dark gray bars); saline 5 ml/kg (not shown, control/control as control, 100% for comparison)) given subcutaneously at 10 min increased intra-abdominal pressures-time. Means ± SD, *P˂0.05, vs. control.

**FIGURE 3:**
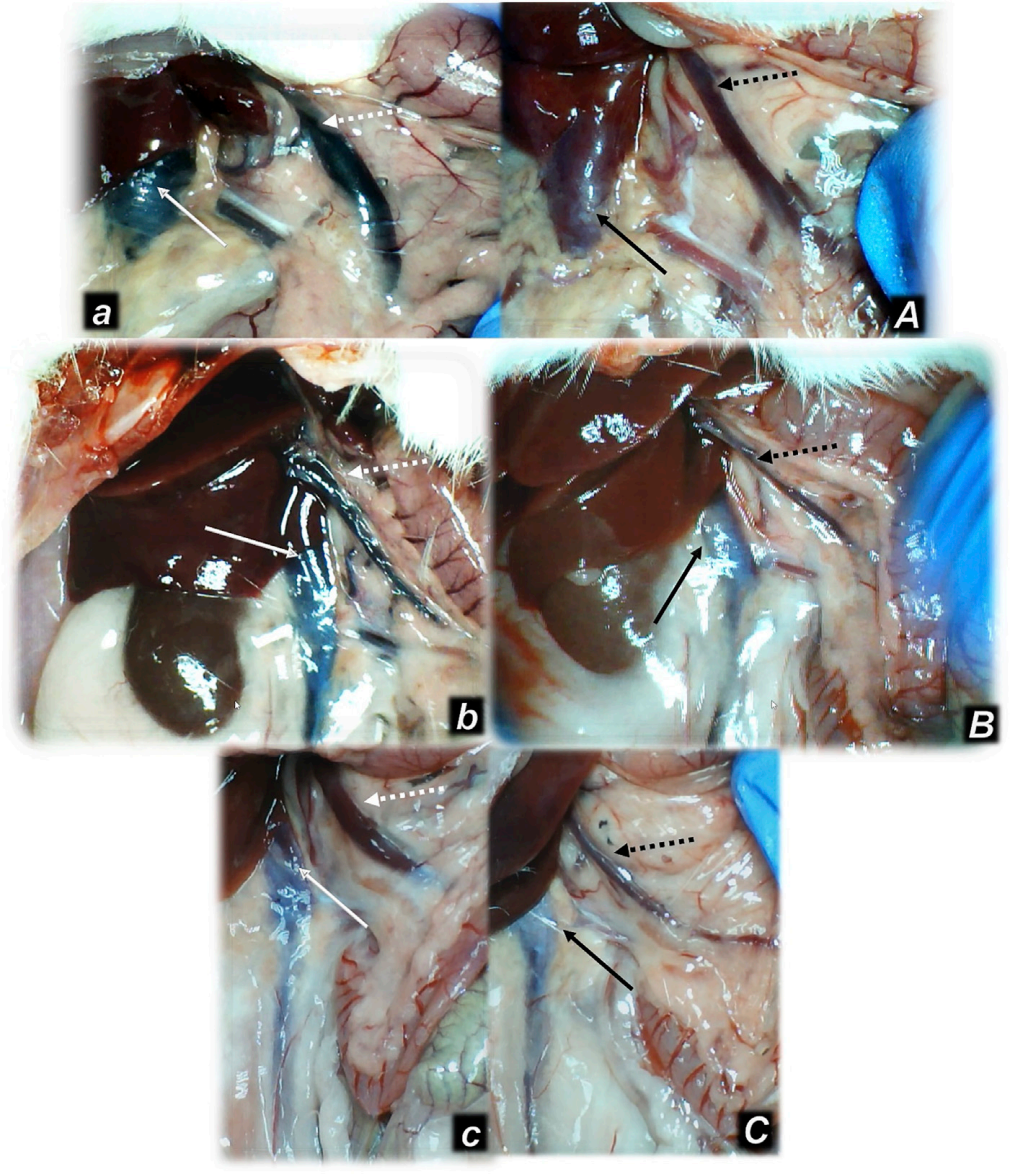
Illustrative presentation of the inferior caval vein (full arrows) and superior mesenteric vein (dashed arrows) after the increased intraabdominal pressure and medication (sc) (saline (5 ml/kg) (white arrows, small letters, congested veins *a, b, c*) or BPC 157 (10 ng/kg) (black arrows, capitals, non-congested veins *A, B, C*): 25 mmHg (60 min) (*a, A*), 40 mmHg (30 min) (*b, B*), and 50 mmHg (30 min) (*c, C*). A camera attached to a VMS-004 Discovery Deluxe USB microscope (Veho, United States).

**FIGURE 4:**
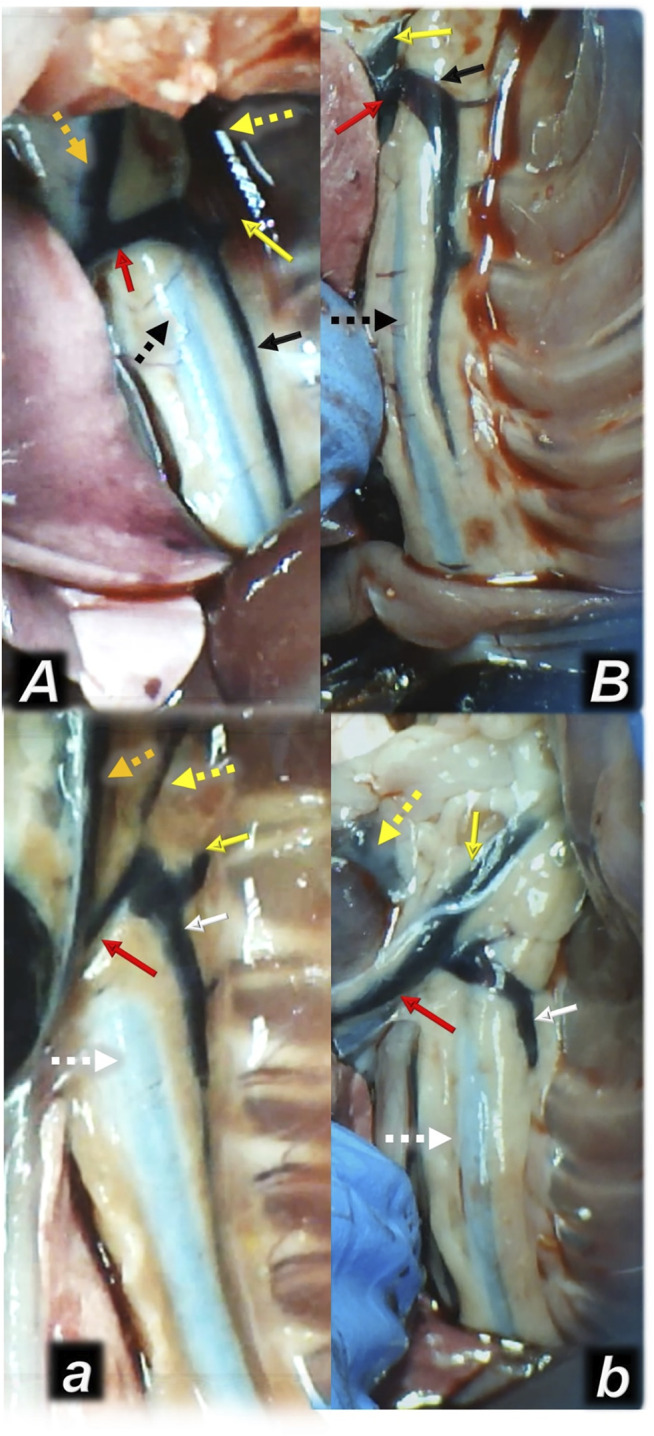
Illustrative presentation of the azygos veins after the increased intraabdominal pressure and medication (sc) (full white arrow, saline (5 ml/kg, low, poor azygos vein presentation a, b) or BPC 157 (full black arrow, 10 ng/kg, upper, functioning azygos vein A, B): 40 mmHg (30 min) (a, A) and 50 mmHg (25 min) (b, B). Aorta (dashed arrows (white (control), black (BPC 157), axillar vein (full yellow arrow), left superior caval vein (red arrow), eternal jugular vein (dashed yellow arrow), internal jugular vein (dark yellow dashed arrow). A camera attached to a VMS-004 Discovery Deluxe USB microscope (Veho, United States).

**FIGURE 5:**
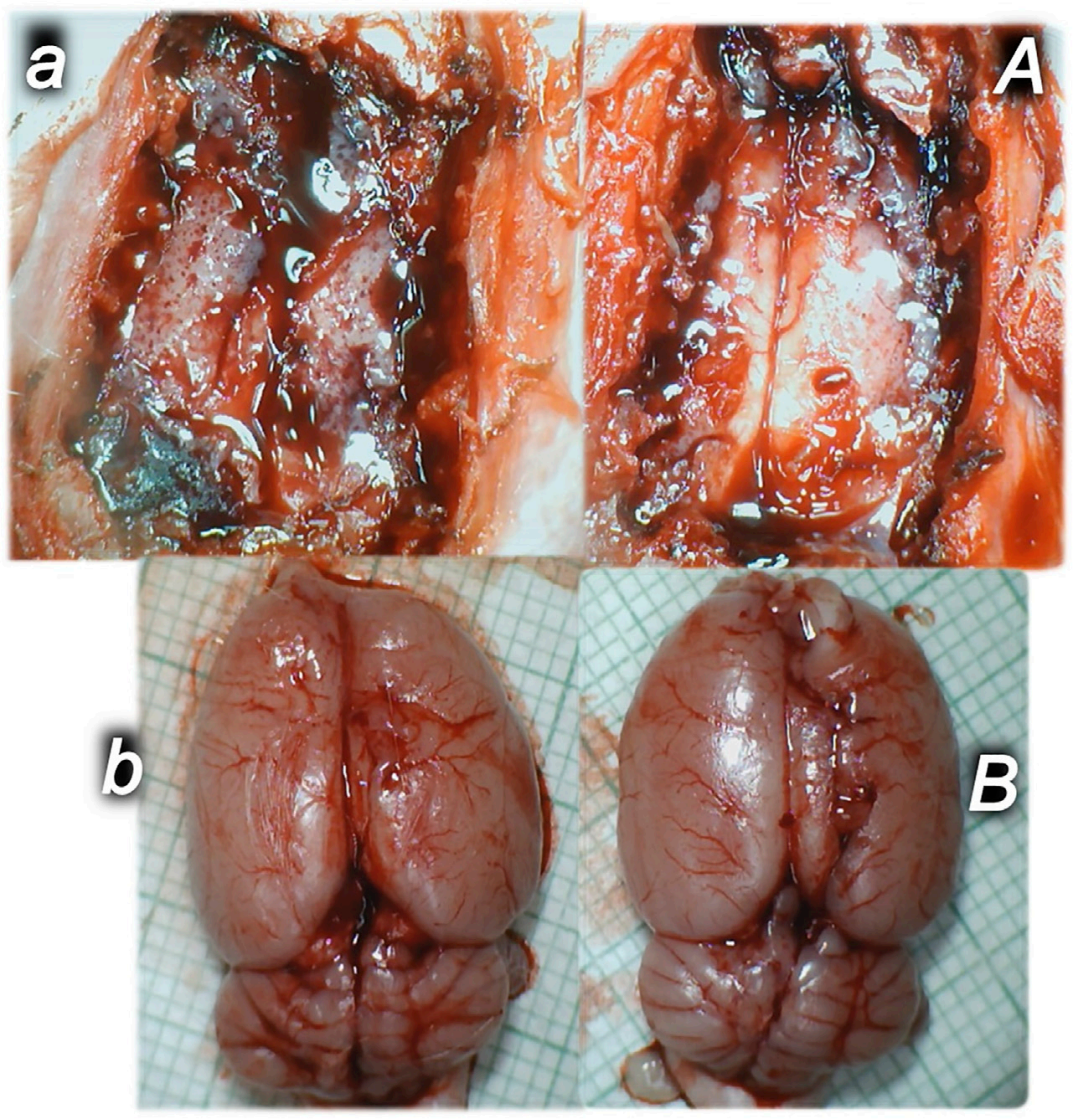
Illustrative brain presentation in the rats with the increased intra-abdominal pressure (50 mm Hg). In calvarial window **(upper)**, at 15 min increased pressure time and medication saline (5 ml/kg ip) **(upper, left, control, *a*)** or BPC 157 (10 ng/kg sc) **(upper, right, *A*)**, at 10 min increased intra-abdominal pressure time. After sacrifice **(low)**, at the 25 min increased intra-abdominal pressure time (saline (5 ml/kg ip) **(low, left, control, *b*)** or BPC 157 (10 ng/kg sc) **(low, right, *B*)** at 10 min increased intra-abdominal pressure time. Prominent brain swelling in control rats **(left)**, completely reversed in BPC 157 rats **(right)**. A camera attached to a VMS-004 Discovery Deluxe USB microscope (Veho, United States).

As a follow-up to the attenuation of blood pressure disturbances, peripherally and centrally, there was a reduction in blood stasis by activating the collateral pathway to compensate for major vessel occlusion due to mechanical compression. Consequently, there were particular effects of BPC 157 on the relative volume of the vessels and brain that may be indicative of the activated defensive response (Figures 2, 3, 4, 5). BPC 157 may decrease the relative volume of the superior mesenteric vein and inferior caval vein and brain (Figures 2, 4, 5). These veins appeared congested (Figures 3, 4), likely due to failed vessels and trapped blood volume (note that the liver and spleen relative weights were increased, along with hemorrhagic lesions in the stomach) (Figures 9, 10) (Figures 3, 4, 5). Evidently, as a particular effect on blood vessels, congestion was reduced by activating the collateral bridging pathway, i.e., the azygos vein (Figure 2), as BPC 157 increased the azygos vein relative volume (Figures 2, 4). In this way, BPC 157 combined the inferior caval vein and left superior caval vein to reestablish blood flow. Finally, regarding brain swelling and increased volume (associated with considerable brain injuries) (Figures 2, 5), BPC 157 rapidly induced a considerable decrease toward normal brain presentation (Figures 2, 5).

### Thrombosis

**FIGURE 6:**
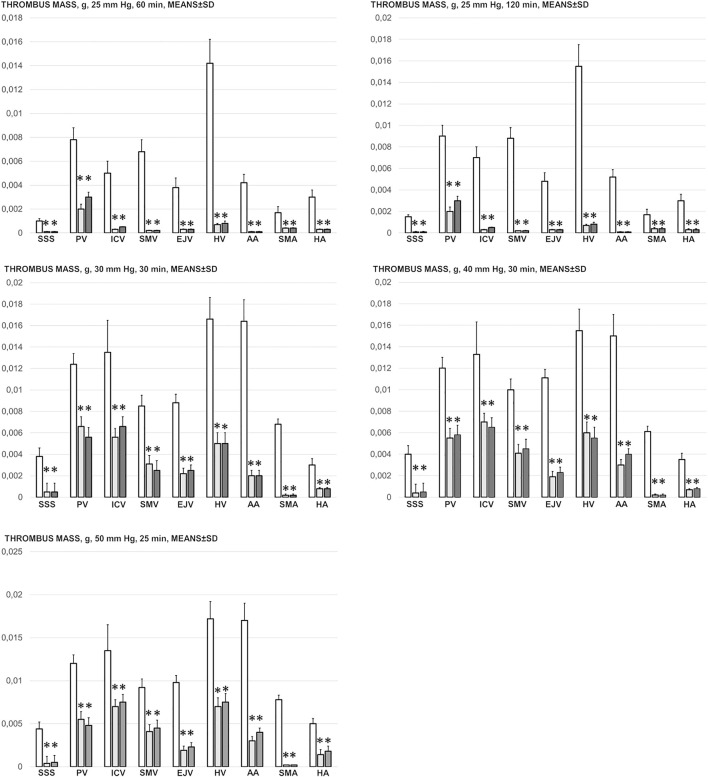
Thrombus mass, g (in the superior sagittal sinus (SSS), portal vein (PV), inferior caval vein (ICV), superior mesenteric vein (SMV), external jugular vein (EJV), hepatic veins (HV), abdominal aorta (AA), superior mesenteric artery (SMA) and hepatic artery (HA)) in the thiopental-anesthetized rats with the increased intra-abdominal pressures at 50 mmHg for 25 min, at 30 mmHg or 40 mmHg for 30 min, at 25 mmHg for 60 min increased intra-abdominal pressures-time and in the esketamine-anesthetized rats with the increased intra-abdominal pressures at 25 mmHg for 120 min increased intra-abdominal pressures-time, following medication (BPC 157 10 μg/kg (light gray bars), 10 ng/kg (dark gray bars); saline 5 ml/kg (white bars)) given subcutaneously at 10 min increased intra-abdominal pressures-time. Means ± SD, *P˂0.05, vs. control.

Likewise, in the cause-consequence course of the therapy, BPC 157 reduced thrombosis, both peripherally and centrally. Without therapy, thrombosis imminently occurred along with high intra-abdominal pressure, peripherally in veins (i.e., portal vein and inferior caval vein, superior mesenteric vein, hepatic veins, and external jugular vein) and in arteries (i.e., superior mesenteric artery, hepatic artery and abdominal aorta) and centrally (i.e., superior sagittal sinus) (Figure 6). Note that, without therapy, while thrombosis was present in all investigated vessels, with an initial increase of 25 mm, the most prominent clots appeared in the hepatic veins. With further pressure increases (30, 40, and 50 mmHg), clot formation generally increased, and prominent clots also appeared in the portal vein and inferior caval vein and in the abdominal aorta.

### Heart and ECG Disturbances

Commonly, high intra-abdominal pressures were timely along with the nodal rhythm, with dominant ST-elevation and bradycardia. Extreme bradycardia and asystole appeared as the ultimate outcome, at 20 ± 2 min (50 mmHg), 25 ± 5 min and 28 ± 2 min (30 mmHg and 40 mmHg), and 55 ± 8 min (25 mmHg) in control rats under thiopental anesthesia and at 110 ± 25 min in esketamine-anesthetized control rats. However, the evidence shows that despite continuously maintaining high intra-abdominal pressure, in all BPC 157-treated rats, heart function was consistently maintained, with fewer ECG disturbances. The sinus rhythm was preserved, with occasional first-degree AV block, but with no ST-elevation. Extreme bradycardia and asystole were not observed. This occurred along with normal heart microscopic presentation, unlike the myocardial congestion and sub-endocardial infarction observed in controls (Figure 11).

### Gastrointestinal, Lung, Liver, Kidney, and Heart Lesions

**FIGURE 7:**
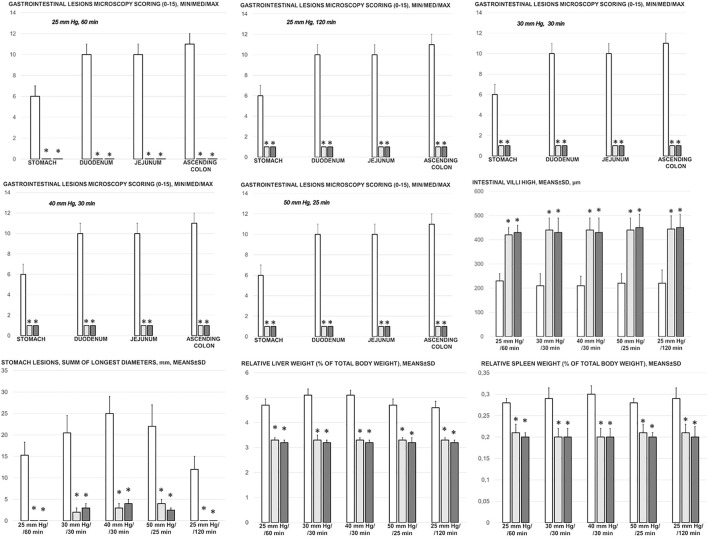
Gastrointestinal lesions microscopy scoring (0–15), stomach, duodenum, jejunum, ascending colon and intestinal villi high, µm, and stomach lesions (sum of longest lesions diameters, mm), relative liver weight (% of total body weight), relative spleen weight (% of total body weight) in the thiopental-anesthetized rats with the increased intra-abdominal pressures at 50 mmHg for 25 min, at 30 mmHg or 40 mmHg for 30 min, at 25 mmHg for 60 min increased intra-abdominal pressures-time, and in the esketamine-anesthetized rats with the increased intra-abdominal pressures at 25 mmHg for 120 min increased intra-abdominal pressures-time, following medication (BPC 157 10 μg/kg (light gray bars), 10 ng/kg (dark gray bars); saline 5 ml/kg (white bars)) given subcutaneously at 10 min increased intra-abdominal pressures-time. Minimum (min), maximum (max), median (med), means ± SD, *P˂0.05, vs. control.

**FIGURE 8:**
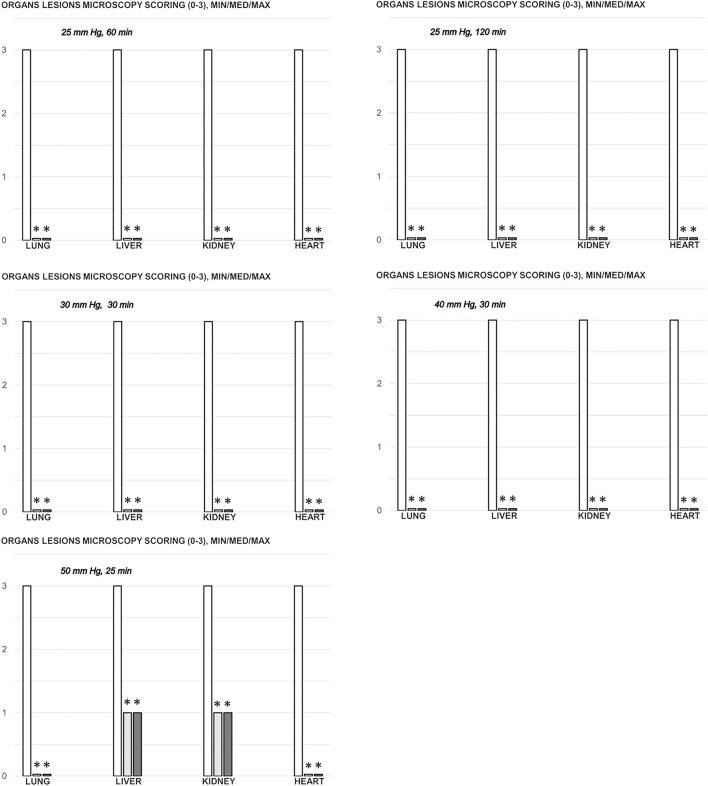
Organs (lung, liver, kidney, heart) microscopy scoring (0–3), in the thiopental-anesthetized rats with the increased intra-abdominal pressures at 50 mmHg for 25 min, at 30 mmHg or 40 mmHg for 30 min, at 25 mmHg for 60 min increased intra-abdominal pressures-time, and in the esketamine-anesthetized rats with the increased intra-abdominal pressures at 25 mmHg for 120 min increased intra-abdominal pressures-time, following medication (BPC 157 10 μg/kg (light gray bars), 10 ng/kg (dark gray bars); saline 5 ml/kg (white bars)) given subcutaneously at 10 min increased intra-abdominal pressures-time. Minimum (min), maximum (max), median (med), *P˂0.05, vs. control. Figure 9. Illustrative presentation of gross and microscopic presentation. Gross presentation. Stomach (*a, A*) and liver (*b,B*) (white letters) after the increased intraabdominal pressure and medication (sc) (saline (5 ml/kg, left, stomach and duodenum with multiple small hemorrhagic lesions (*a*), and congested liver (*b*) presentation) or BPC 157 (10 ng/kg, right, stomach and duodenum, and liver *A, B*): 25 mmHg (30 min) (*a, A*) and 40 mmHg (30 min) (*b, B*). A camera attached to a VMS-004 Discovery Deluxe USB microscope (Veho, United States). Microscopy presentation. Stomach (*a, A*) and colon (*b, B*) (black letters) presentation in rats with the increased intra-abdominal pressure at 50 mmHg for 25 min treated at 10 min increased intra-abdominal pressure time with saline (control, *a, b*) or BPC 157 (*A, B*). The control group showed marked hyperemia and congestion of the stomach wall (*a*) and a reduction of the colonic crypts with focal denudation of the superficial epithelia (*b*). BPC 157-treated rats exhibit presentation close to normal gastrointestinal tract presentation (*A, B*). (HE; *a, A*, magnification ×100, scale bar 200 μm; *b, B*, magnification ×200, scale bar 100 μm).

**FIGURE 9:**
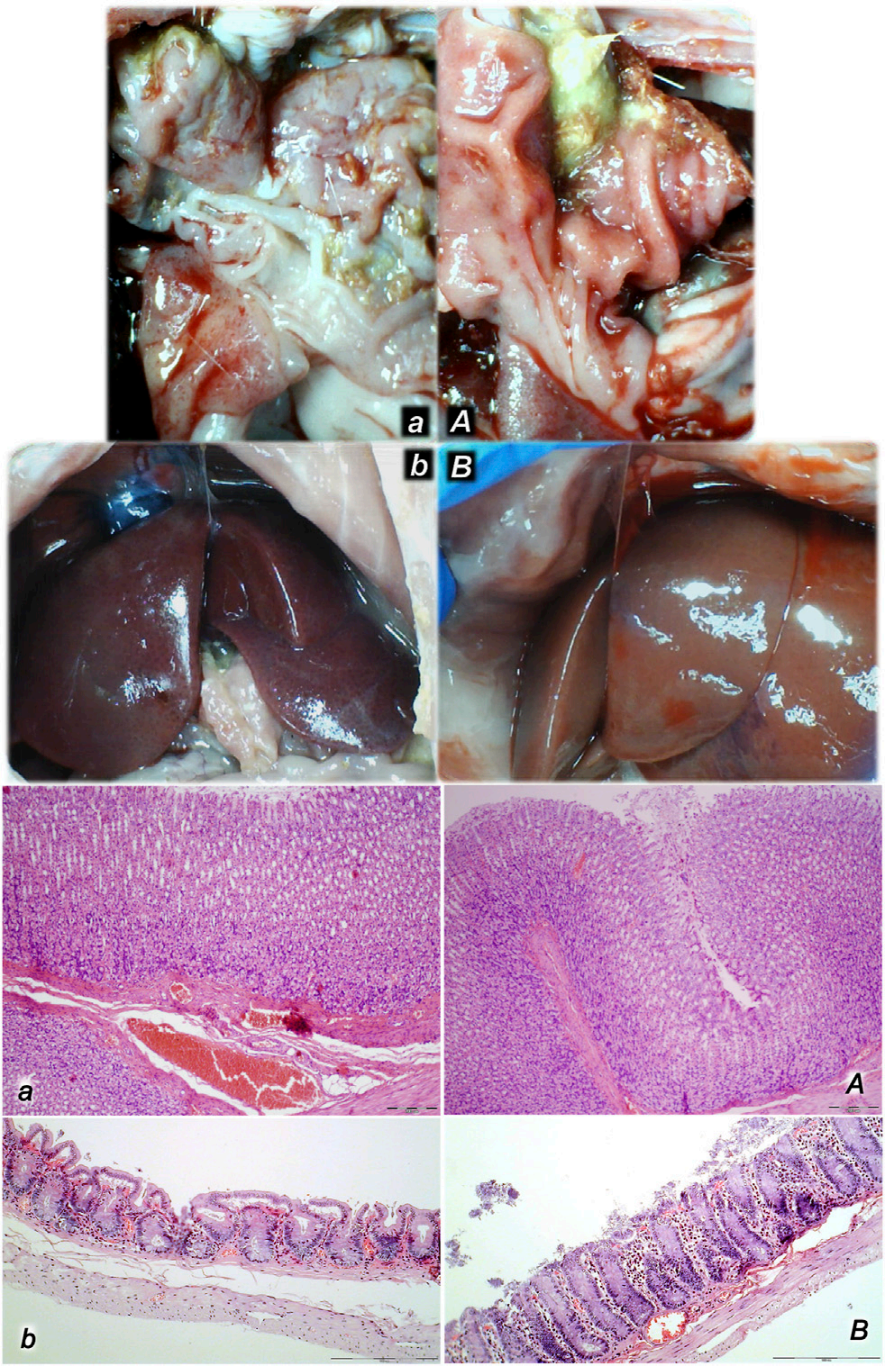
Illustrative presentation of gross and microscopic presentation. Gross presentation. Stomach (a, A) and liver (b,B) (white letters) after the increased intraabdominal pressure and medication (sc) (saline (5 ml/kg, left, stomach and duodenum with multiple small hemorrhagic lesions (a), and congested liver (b) presentation) or BPC 157 (10 ng/kg, right, stomach and duodenum, and liver A, B): 25 mmHg (30 min) (a, A), and 40 mmHg (30 min) (b, B). The camera attached to a VMS-004 Discovery Deluxe USB microscope (Veho, United States). Microscopy presentation. Stomach (a, A) and colon (b, B) (black letters) presentation in rats with the increased intra-abdominal pressure at 50 mmHg for 25 min treated at 10 min increased intra-abdominal pressure time with saline (control, a, b) or BPC 157 (A, B). The control group showed marked hyperemia and congestion of the stomach wall (a) and a reduction of the colonic crypts with focal denudation of the superficial epithelia (b). BPC 157-treated rats exhibit presentation close to normal gastrointestinal tract presentation (A, B). (HE; a, A, magnification ×100, scale bar 200 μm; b, B, magnification ×200, scale bar 100 μm).

**FIGURE 10:**
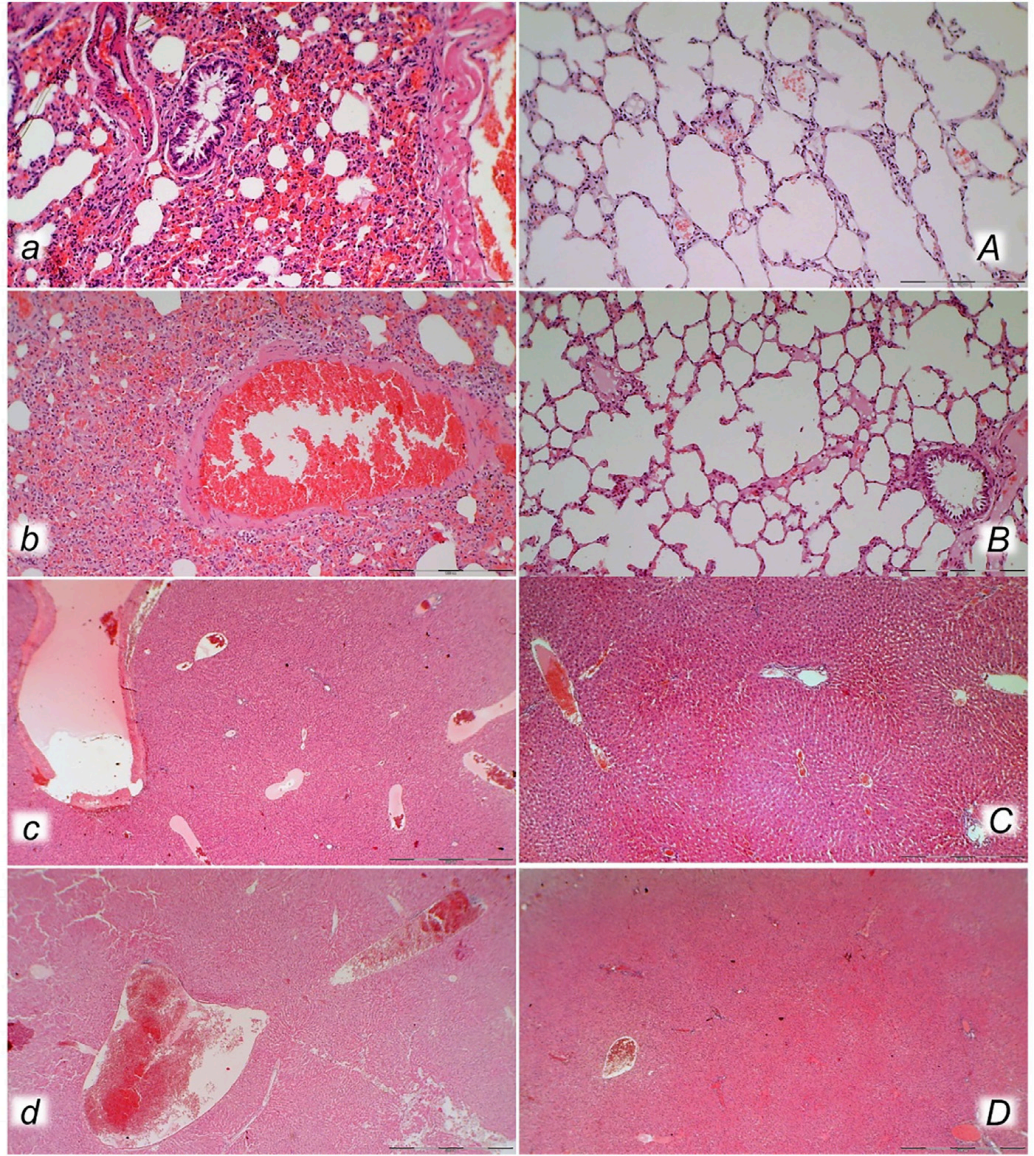
Lung (*a, A, b, B*) and liver (*c, C, d, D*) presentation in rats with the increased intra-abdominal pressure at 25 mmHg for 60 min (*a, A, c, C*) or at 50 mmHg for 25 min (*b, B, d, D*), treated at 10 min increased intra-abdominal pressure time with saline (control, *a, b, c, d*) or BPC 157 (*A, B, C, D*). *a, b.* Lung parenchyma with marked congestion and large areas of intra-alveolar hemorrhage in control rats. *A, B.* Normal lung parenchyma in BPC 157-treated rats. *c, d*. Vascular dilatation of liver parenchyma in controls, normal architecture in BPC 157 treated rats (C) and slight congestion of liver parenchyma (*D*). (HE; magnification ×200, scale bar 100 μm (*a, A, b, B*)*;* magnification ×100, scale bar 500 μm (*c, C, d, D*)).

**FIGURE 11:**
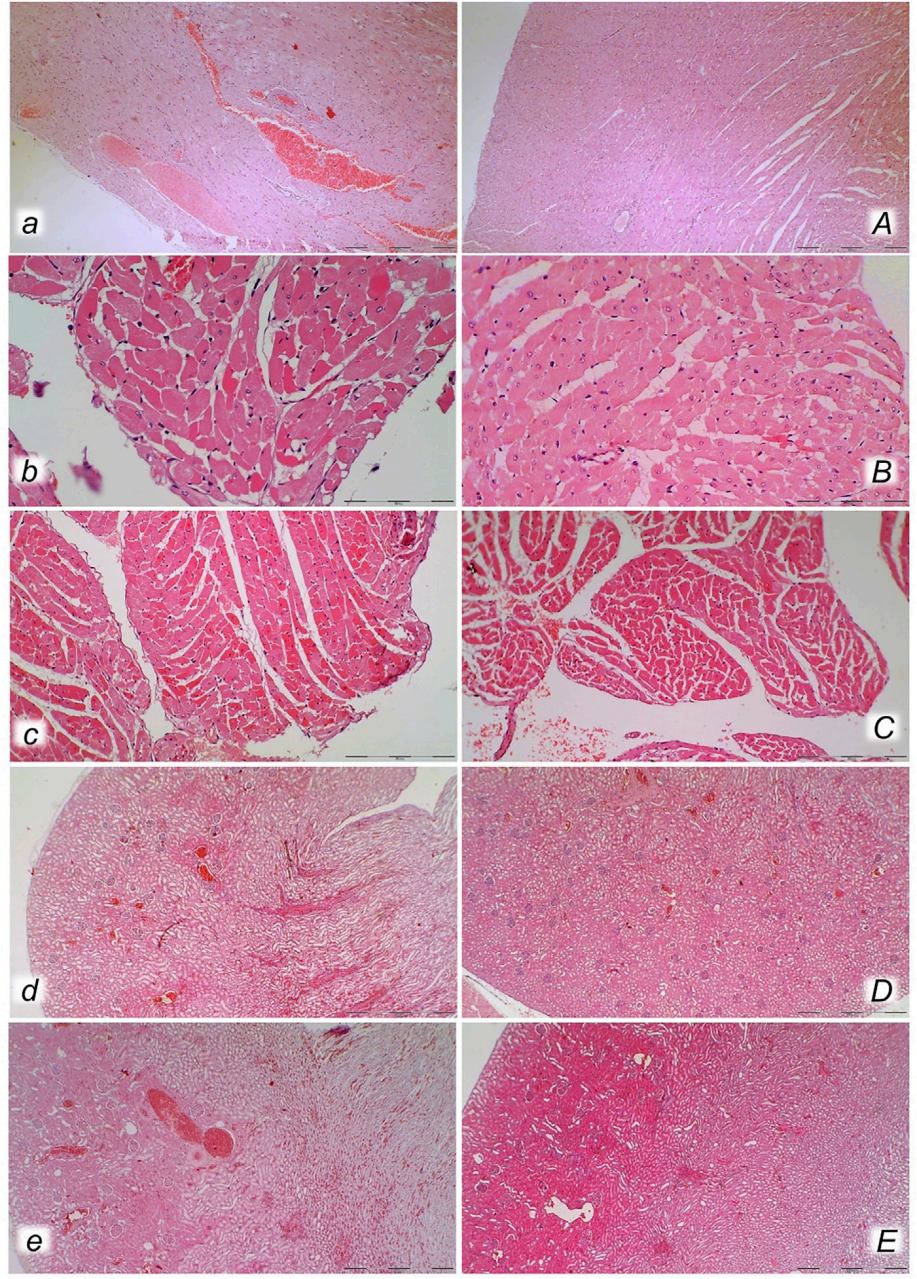
Heart (*a, A, b, B, c, C*) and kidney (*d, D, e, E*) presentation in the rats with the increased intra-abdominal pressure at 25 mmHg for 60 min (*a, A, b, B, d, D*) or at 50 mmHg for 25 min (*c, C, e, E*), treated at 10 min increased intra-abdominal pressure time with saline (control, *a, b, c, d, e*) or BPC 157 (*A, B, C, D, E*). Marked congestion of myocardium of control rats, with subendocardial infract found in all control rats at 25 mmHg (*a, b*), and at 50 mmHg of intra-abdominal pressure (*c*), while myocardium was preserved in all BPC 157- treated rats (*A, B, C*). Severe congestion of renal tissue was found in control rats at 25 mmHg (*d)* and at 50 mmHg of intra-abdominal pressure (*e*), while in BPC 157- treated rats, no changes were found at 25 mmHg intra-abdominal pressure (*D*) and only discrete congestion was found at 50 mmHg of intra-abdominal pressure (*E*). (HE; magnification ×200, scale bar 100 μm (*a, A*); x400, scale bar 50 μm (*b, B, c, C*); x100, scale bar 500 μm (*d, D, e, E*)).

Consequently, as part of the cause-consequence therapeutic course, i.e., reduced intracranial (superior sagittal sinus), portal, and caval hypertension, reduced aortal hypotension, and activated collateral pathway, BPC 157 reduced the severity of lesions in the gastrointestinal tract and other organs commonly noted in the untreated rats with high intra-abdominal pressures (Figures 7, 8, 9, 10, 11; Supplementary Figures S1, S2).

With an increase in severity from the upper toward the lower part of the gastrointestinal tract, control rats demonstrated transmural hyperemia of the entire gastrointestinal tract, stomach, duodenum, and small and large bowel wall, along with a reduction in the villi in the intestinal mucosa, crypt reduction with focal denudation of superficial epithelia, and dilatation of the large bowel (Figures 7, 8, 9, 10, 11; Supplementary Figures S1, S2). Regularly, in BPC 157-treated rats, we noted no or minimal congestion in the gastrointestinal mucosa with well-preserved intestinal villi and colonic crypts with no dilatation of the large bowel. Considering intra-abdominal hypertension at grade III and grade IV and the therapeutic effect, it was not surprising to find a considerable decrease in villi height in all control rats with high intra-abdominal pressure (Figures 7, 9; Supplementary Figures S1, S2) and preserved villi height in the BPC 157-treated rats (similar to the villi height in healthy rats, indicating preserved intestinal function despite high intra-abdominal pressure).

Without therapy, severe lesions were observed in the rats with high intra-abdominal pressures, characterized by marked congestion of the myocardium and subendocardial infarcts (Figure 11), marked congestion and large areas of intra-alveolar hemorrhage in the lung (Figure 10), vascular dilation of the liver parenchyma (Figure 10), and renal congestion (Figure 11). In contrast, as a result of treatment, the equally high intra-abdominal pressures in BPC 157-treated rats led to only mild congestion in the gastrointestinal tract, liver, and kidney (Figures 7, 8, 9, 10, 11), particularly with high intra-abdominal pressures at 40 and 50 mmHg (otherwise, no changes in the liver and renal parenchyma were observed). The myocardium was preserved, with no change in the lung parenchyma (Figure 8, 10, 11).

### Brain Lesions, Cerebral and Cerebellar Cortex, Hypothalamus/Thalamus, and Hippocampus

Without therapy, the consistently downhill course of intra-abdominal hypertension in rats with high intra-abdominal pressures led to multiple organ lesions, widespread thrombosis, disturbed ECG and blood pressure, portal and caval hypertension, aortal hypotension, and, in particular, intracranial (superior sagittal sinus) hypertension (Figures 1–15) along with severe brain lesions (Figures 12, 13, 14, 15). Moreover, evidently, the brain was consistently swollen (Figures 1, 5), resulting in brain damage in all investigated areas (Figures 12, 13, 14, 15).

**FIGURE 12:**
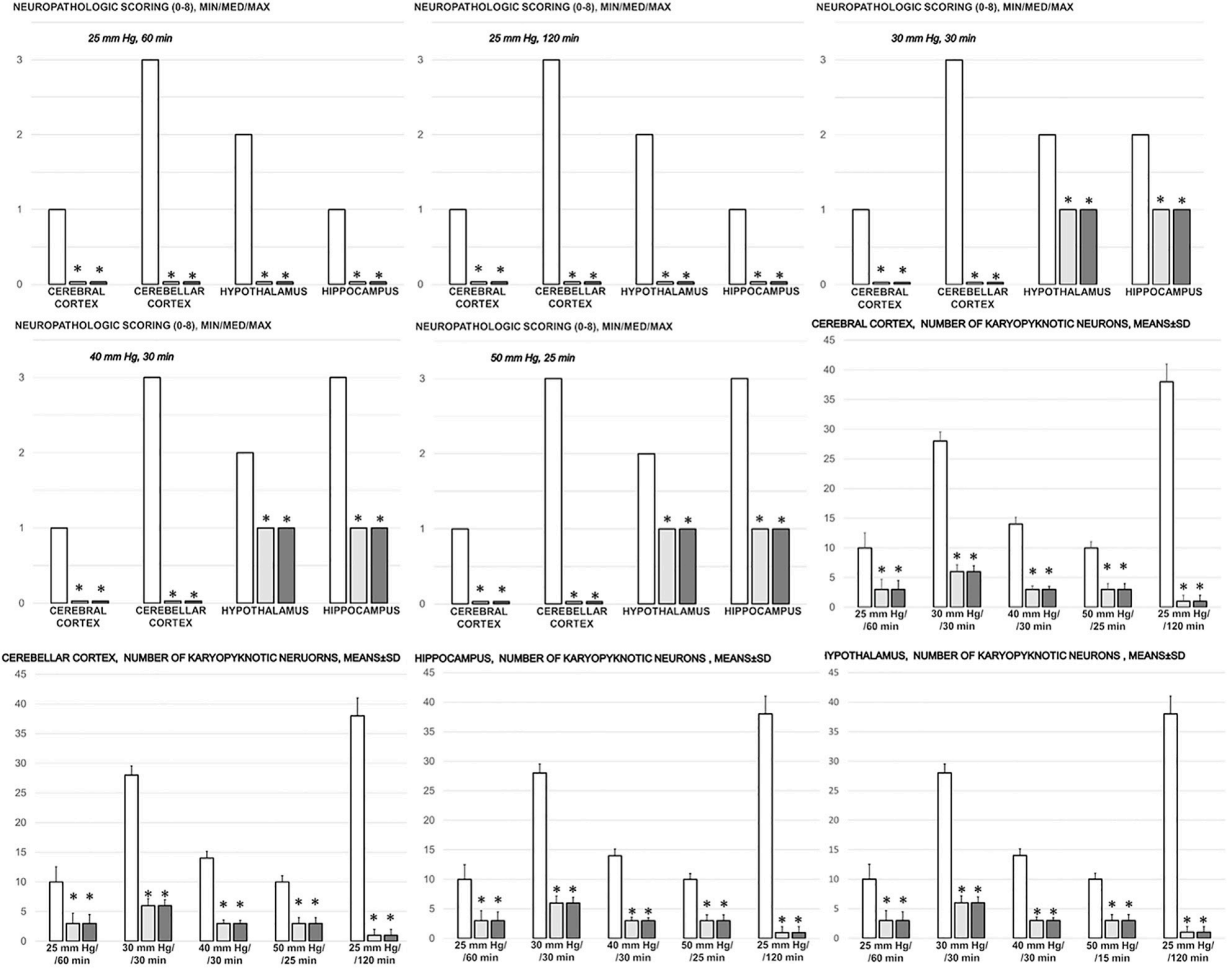
Neuropathologic scoring (0–8), cerebral cortex, cerebellar cortex, hypothalamus, hippocampus, and the number of karyopyknotic cells, cerebral cortex, cerebellar cortex, hypothalamus, hippocampus, in the thiopental-anesthetized rats with the increased intra-abdominal pressures at 50 mmHg for 25 min, at 30 mmHg or 40 mmHg for 30 min, at 25 mmHg for 60 min increased intra-abdominal pressures-time, and in the esketamine-anesthetized rats with the increased intra-abdominal pressures at the 25 mmHg at the 120 min increased intra-abdominal pressures-time, following medication (BPC 157 10 μg/kg (light gray bars), 10 ng/kg (dark gray bars); saline 5 ml/kg (white bars)) given subcutaneously at 10 min increased intra-abdominal pressures-time. Minimum (min), maximum (max), median (med), means ± SD, *P˂0.05, vs. control.

**FIGURE 13:**
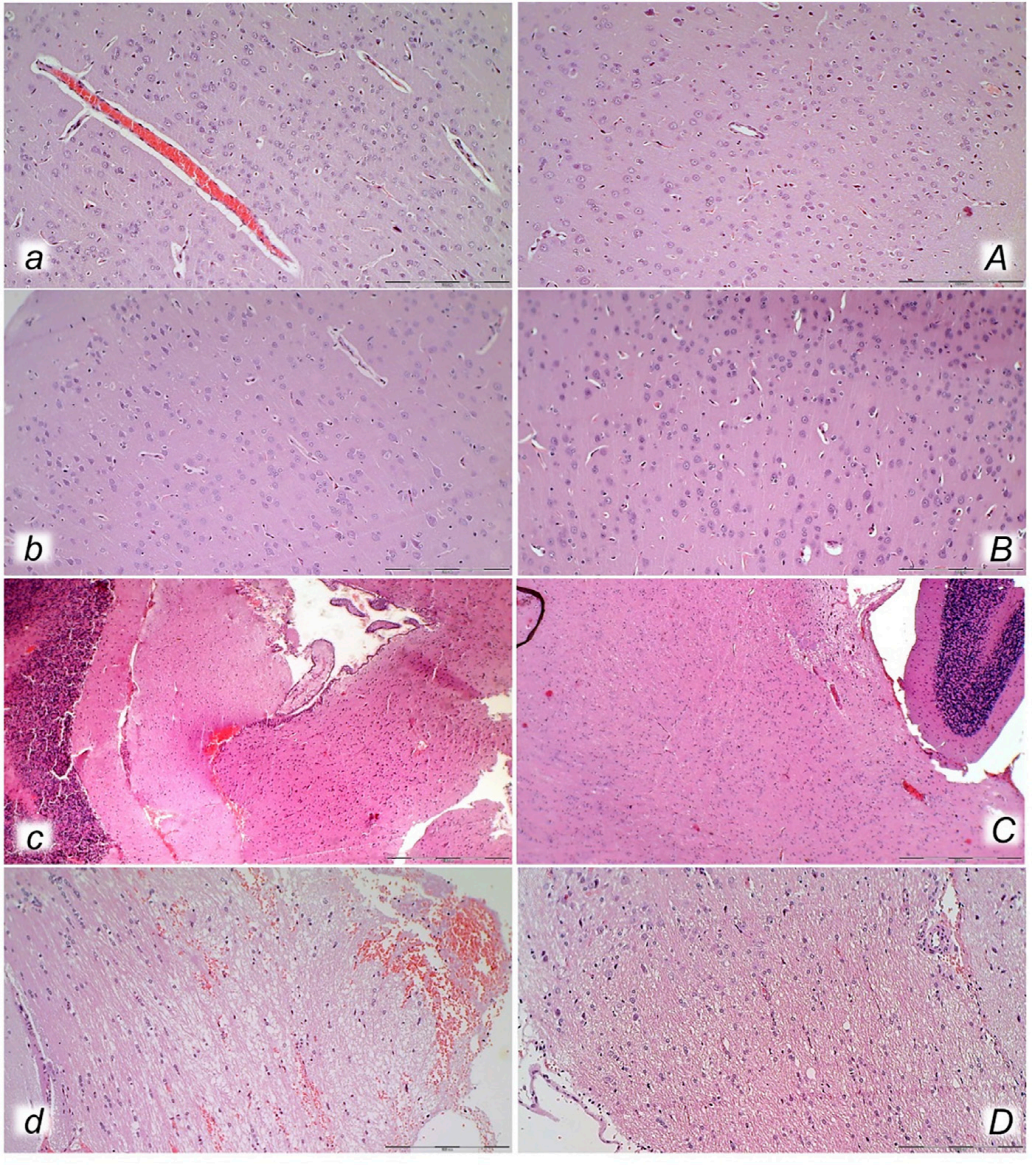
Neuropathological changes of the cerebral cortex (*a, A, b, B*), cerebellar cortex (*c, C*) and pons (*d, D*) in rats with the increased intra-abdominal pressure at 25 mmHg for 60 min (*a, A, c, C*) or at 50 mmHg for 25 min (*b, B, d, D*), treated at 10 min increased intraabdominal pressure time with saline (control, *a, b, c, d*) or BPC 157 (*A, B, C, D*). Generalized edema and congestion (*a, b, c, d*) with an increased number of karyopyknotic cells were found in the cerebral cortex (*a, b*) that was significantly different from the cortex area in BPC 157-treated rats (*A, B*). In control rats, intracerebral hemorrhage was found in infratentorial space (*d*), mostly in cerebellopontine angle/area (*c*) with generalized edema and congestion of central nervous system, while no hemorrhage (*C*) and only mild edema was found in treated animals, mostly at 50 mmHg intra-abdominal pressure (*D*). (HE; magnification ×200, scale bar 100 μm (*a, A, b, B, d, D*)*;* magnification ×100, scale bar 200 μm (*c, C*)).

**FIGURE 14:**
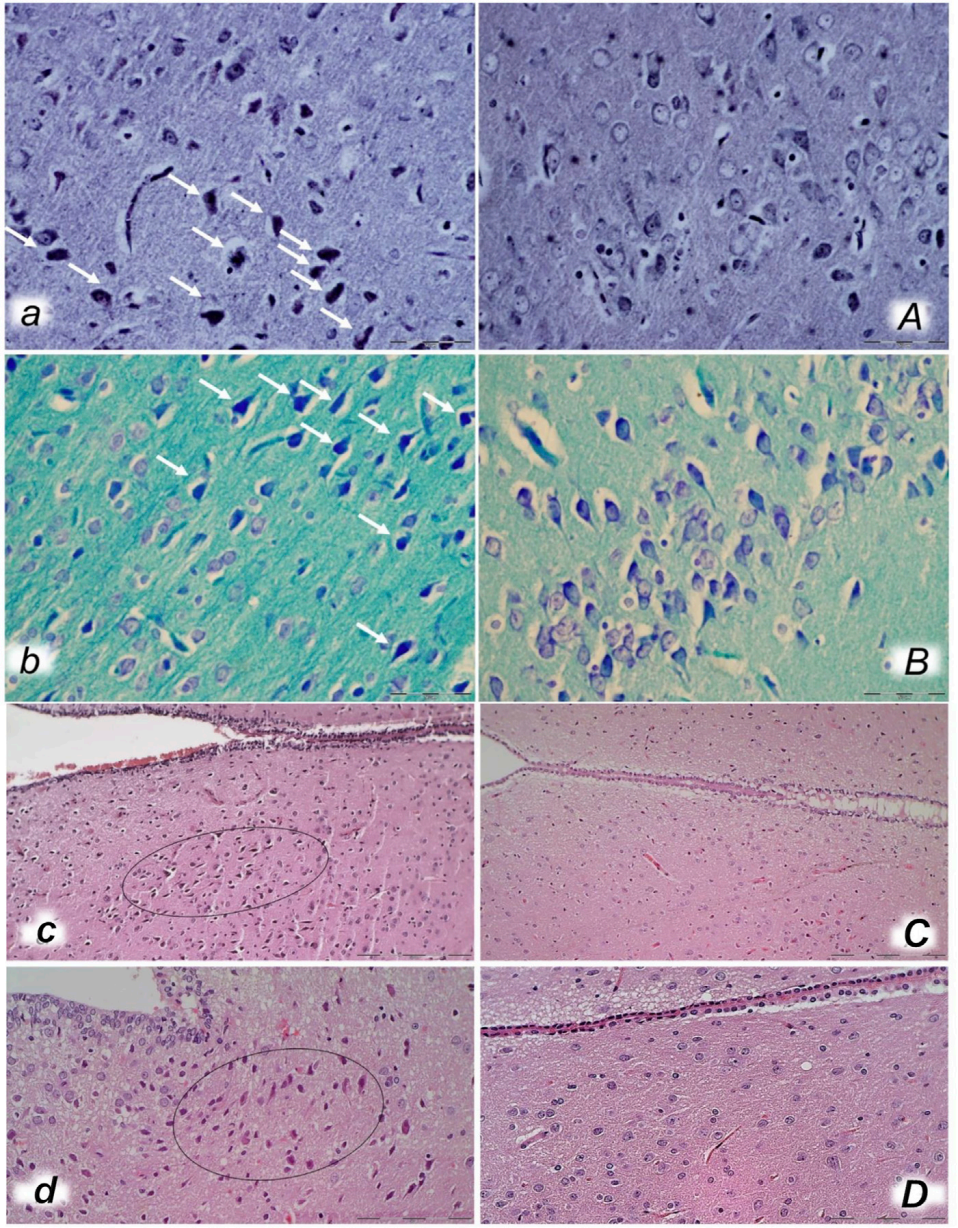
Bielschowsky and Klüver–Barrera histochemical staining presenting neuropathological changes of cerebral cortex in rats with the increased intra-abdominal pressure at 30 mmHg for 30 min (*a, A, b, B*) treated at 10 min increased intraabdominal pressure time with saline (control *a, b*) or BPC 157 (*A, B*). In control rats, an increased number of karyopyknotic cells was found in the cerebral cortex (white arrows) (*A, B*) that was significantly different from the cortex area in BPC 157-treated rats (*a, b*). (Bielschowsky staining (*a, A*); Klüver–Barrera staining (*b, B*); magnification ×600, scale bar 50 μm). Neuropathological changes of hypothalamic/thalamic area (*c, C, d, D*) presentation in rats with the increased intra-abdominal pressure at 25 mmHg for 60 min (*c, C*) or at 50 mmHg for 25 min (*d, D*), treated at 10 min increased intra-abdominal pressure time with saline (control, *c, d*) or BPC 157 (*C, D*). A marked karyopyknosis was found in all control rats (marked in oval) (*c,* 25 mmHg/60 min); *d,* 50 mmHg/25 min) while preserved brain tissue was found in BPC 157-treated rats (*C,* 25 mmHg/60 min); *D,* 50 mmHg/25 min). (HE; magnification ×400, scale bar 50 μm).

**FIGURE 15:**
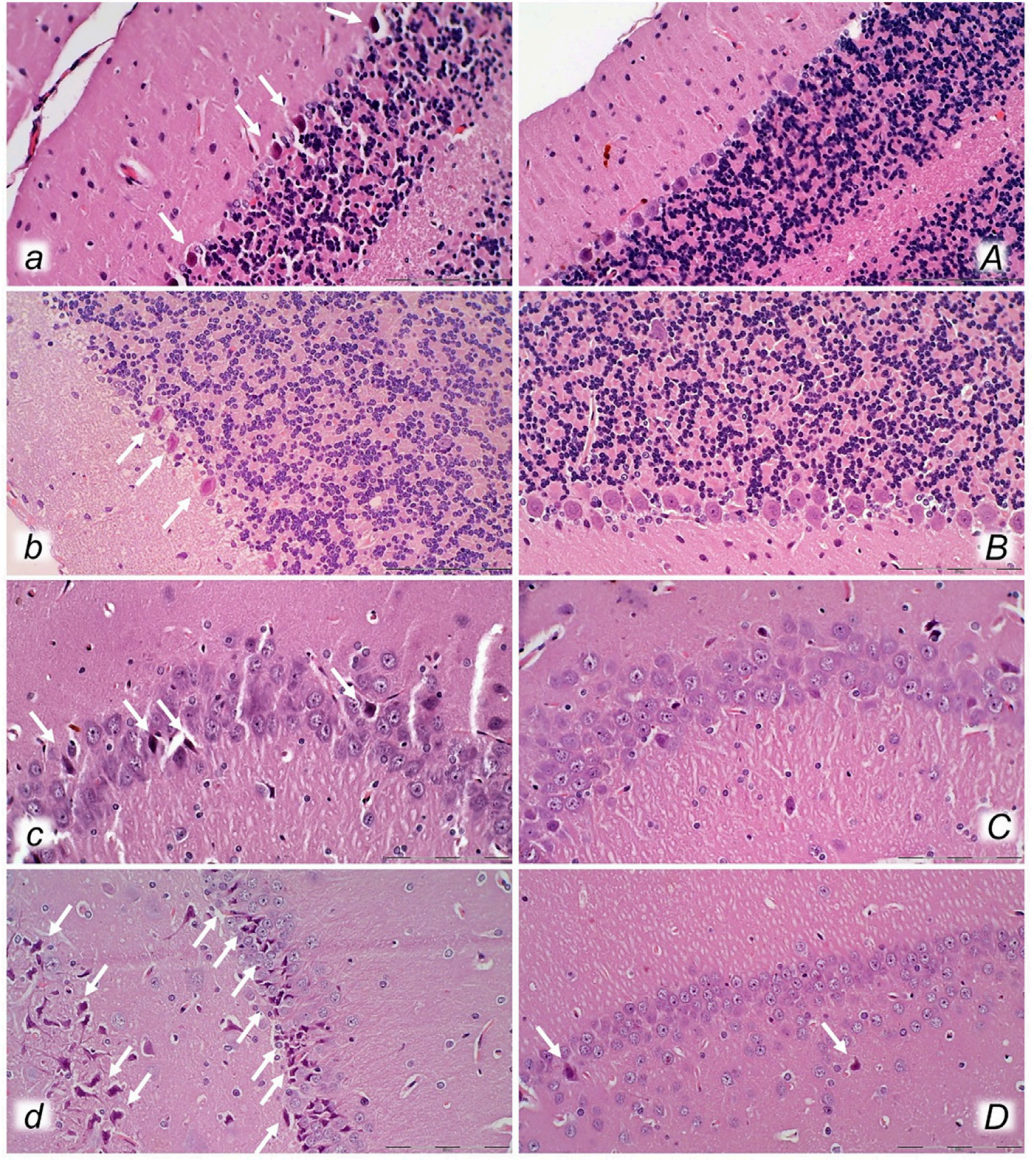
Neuropathological changes of cerebellar cortex (*a, A*, *b, B*) and hippocampus (c*, C, d, D*) in rats with the increased intra-abdominal pressure at 25 mmHg for 60 min (*a, A, c, C*) or at 50 mmHg for 25 min (*b, B, d, D*), treated at 10 min increased intra-abdominal pressure time with saline (control, *a, b, c, d*) or BPC 157 (*A, B, C, D*). Control rats exhibited within cerebellar area karyopyknosis and degeneration of Purkinje cells (*a, b*). Marked and progressive karyopyknosis and degeneration of pyramidal cell of the hippocampus was observed in control rats (arrows) at 25 mmHg intraabdominal pressure (*c*) and even more at 50 mmHg intra-abdominal pressure (*d*). No change was found in the cerebellar and hippocampal area in BPC 157- treated rats at 25 mmHg intra-abdominal pressure (*A, B, C*) and only rare hippocampal karyopyknotic cells (arrows) at 50 mmHg intra-abdominal pressure (*D*) (HE; magnification ×400, scale bar 50 μm).

In general, congestion of the cerebral and cerebellar cortex, hypothalamus/thalamus, and hippocampus was observed, with edema and large areas with increased numbers of karyopyknotic cells, as well as intracerebral hemorrhage, mostly in the infratentorial space, affecting the cerebello angle/area (Figures 12, 13, 14, 15). We noted an increased number of karyopyknotic cells in all four regions, i.e., the cerebral and cerebellar cortex, hippocampus, and hypothalamus/thalamus (Figure 14). Especially, there was karyopyknosis and degeneration of Purkinje cells of the cerebellar cortex and marked karyopyknosis of pyramidal cells in the hippocampus. In particular, these brain lesions appeared to be distinctively affected by high intra-abdominal pressure; i.e., the most progressive hippocampal neuronal damage was found with the highest intra-abdominal pressure. Contrarily, as a cause-consequence of BPC 157 therapy, i.e., reduced intracranial (superior sagittal sinus) hypertension and gross brain swelling, along with reduced portal and caval hypertension, aortal hypotension, abrogated thrombosis, and an activated collateral pathway, these lesions were largely reduced in BPC 157-treated rats, with a highly protected cortex, hypothalamus/thalamus, and hippocampus, as well as healthy Purkinje cells in the cerebellar cortex. BPC 157-treated rats showed a few karyopyknotic neuronal cells in the analyzed neuroanatomic structures.

Quantitative analysis of neuronal damage in the karyopyknotic areas in all four neuroanatomic structures showed no or only a few karyopyknotic neural cells (Figure 12). The white matter was more vulnerable to chronic cerebral injury. No white matter lesions were found in both groups of animals using modified Bielschowsky silver staining and Klüver–Barrera staining.

In summary, after BPC 157 therapy, rats with high intra-abdominal pressures (grade III and grade IV) exhibited markedly attenuated portal and caval hypertension, ameliorated aortal hypotension, and markedly attenuated superior sagittal sinus hypertension. Additionally, venous and arterial thrombosis was attenuated, both peripherally and centrally, which markedly mitigated stasis and moreover reduced brain, heart, lung, liver, kidney, and gastrointestinal lesions as the untreated result. These reductions were ascribed to the key finding of an activated particular collateral pathway, i.e., the azygos vein, which combined the inferior caval vein and left superior vein to reorganize blood flow.

## Discussion

We investigated the reversal of abdominal compartment syndrome induced by the stable gastric pentadecapeptide BPC 157 due to its previously observed therapeutic effect noted in vessel occlusion syndromes (Vukojevic et al., 2018; Gojkovic et al., 2020; Kolovrat et al., 2020; Gojkovic et al., 2021a; Knezevic et al., 2021a; Knezevic et al., 2021a; Gojkovic et al., 2021b; Knezevic et al., 2021b; Strbe et al., 2021).

With the applied procedure (i.e., 25, 30, 40, or 50 mmHg intra-abdominal hypertension), there was a regular downhill chain of events, regardless of the type of anesthesia (i.e., esketamine, as ketamine is an antioxidant (Xingwei et al., 2014) that may provide a more prolonged survival period than thiopental). The abdominal wall compliance threshold was crossed mechanically, with no further stretch of the abdomen; this increased intra-abdominal pressure, compressed vessels and organs, and pushed up the diaphragm as a predetermined definitive outcome (Depauw et al., 2019). Abdominal compartment syndrome appeared as a multiple occlusion syndrome that could not be avoided unless therapy was given. Regularly, reciprocal changes in the abdominal, thoracic, and brain cavities (Depauw et al., 2019) rapidly appeared as determinants of vascular failure. Therefore, in the rats with intra-abdominal hypertension, multiorgan failure (i.e., gastrointestinal, brain, heart, liver, and kidney lesions), portal and caval hypertension, aortal hypotension, intracranial (superior sagittal sinus) hypertension, and generalized thrombosis appeared. This led to generalized stasis, generalized Virchow triad presentation, and severe ECG disturbances; therapy was able to provide adequate compensation (i.e., activation of collateral pathways to reestablish blood flow), both rapid and sustained, as demonstrated with BPC 157 therapy. As a prime and practical confirmation, rats with major vessel ligation and occlusion, in either artery and/or vein, and either peripherally or centrally, exhibited a similar syndrome (Vukojevic et al., 2018; Gojkovic et al., 2020; Kolovrat et al., 2020; Gojkovic et al., 2021a; Knezevic et al., 2021a; Knezevic et al., 2021a; Knezevic et al., 2021b). Thus, there may be a shared inability to react, leading to innate vascular failure upon major vessel occlusion (ligation) (Vukojevic et al., 2018; Gojkovic et al., 2020; Kolovrat et al., 2020; Gojkovic et al., 2021a; Knezevic et al., 2021a; Knezevic et al., 2021a; Knezevic et al., 2021b) as well as upon the induction of high intra-abdominal pressure, with all vessels compressed. Likewise, with BPC 157 therapy, there may be a shared curative effect, with consistent beneficial evidence in all of the rats with major vessel occlusion (Vukojevic et al., 2018; Gojkovic et al., 2020; Kolovrat et al., 2020; Gojkovic et al., 2021a; Knezevic et al., 2021a; Knezevic et al., 2021a; Knezevic et al., 2021b). Activation of the collateral pathway following occlusion injury fully reduces occlusion syndrome (Vukojevic et al., 2018; Gojkovic et al., 2020; Kolovrat et al., 2020; Gojkovic et al., 2021a; Knezevic et al., 2021a; Knezevic et al., 2021a; Knezevic et al., 2021b). Together, this evidence strongly supports a comparable beneficial effect (i.e., a “bypassing key”) in rats with intra-abdominal hypertension and multiple vessel compression. As a follow-up, fully reduced abdominal compartment syndrome appeared as a confirmative conceptual result.

To reverse abdominal compartment syndrome as a multiple occlusion syndrome disaster, we improved the function of the venous system with the stable gastric pentadecapeptide BPC 157. Considering the multitude of vessels that had been directly compressed, this improvement should be greater than that in specific vessel occlusion syndromes (Vukojevic et al., 2018; Gojkovic et al., 2020; Kolovrat et al., 2020; Gojkovic et al., 2021a; Knezevic et al., 2021a; Knezevic et al., 2021a; Knezevic et al., 2021b) or with an intragastric application of absolute alcohol and intraperitoneal application of lithium overdose, which induce an “occlusion-like” syndrome (Gojkovic et al., 2021b; Strbe et al., 2021). This abdominal compartment syndrome therapy addresses more than one known initial target, i.e., single vessel occlusion (ligation) (Vukojevic et al., 2018; Gojkovic et al., 2020; Kolovrat et al., 2020; Gojkovic et al., 2021a; Knezevic et al., 2021a; Knezevic et al., 2021a; Knezevic et al., 2021b) vs. intragastric application of absolute alcohol (Gojkovic et al., 2021b) and intraperitoneal application of lithium overdose (Strbe et al., 2021) vs. all vessels compressed (increased intra-abdominal hypertension). Thus, by resolving and compensating for damaged functions, the reversal of the chain of harmful consequences of high intra-abdominal pressure can be achieved and abdominal compartment syndrome recovery can occur. Thus, the beneficial findings in rats with severely increased intra-abdominal pressure given the stable gastric pentadecapeptide BPC 157 (for review, see Sikiric et al., 2018) likely occurred due to the effect on compressed essential vessel tributaries, both arterial and venous, peripherally and centrally. As a likely rescue pathway, as seen in the rat Budd–Chiari syndrome (Gojkovic et al., 2020), superior sagittal sinus occlusion syndrome (Gojkovic et al., 2021a), and intragastric application of absolute alcohol (Gojkovic et al., 2021b) or intraperitoneal application of lithium overdose (Strbe et al., 2021), we identified the activated azygos vein pathway and the inferior vena cava–azygos vein–left superior vena cava pathway. The azygos vein pathway was fully activated in BPC 157-treated rats (and thereby provided additional direct blood flow delivery), while it was collapsed in control saline-treated rats with intra-abdominal hypertension.

There may be, however, other activated bypassing loops (Vukojevic et al., 2018; Gojkovic et al., 2020; Kolovrat et al., 2020; Gojkovic et al., 2021a; Knezevic et al., 2021a; Knezevic et al., 2021a; Gojkovic et al., 2021b; Knezevic et al., 2021b). With the harmful effects of intra-abdominal hypertension, peripherally but also centrally, rats with an occluded superior sagittal sinus may be an illustrative example (Gojkovic et al., 2021a). Therefore, we identified central shunts through the ophthalmic vein, angularis vein, facial anterior and posterior veins, and facial vein, as well as the superior cerebral veins, the superior and inferior sinus cavernosus, the sinus petrosus, the sinus transversus, the external jugular vein, the subclavian vein, and the superior vena cava (Gojkovic et al., 2021a). Moreover, with BPC 157 therapy delivered topically to the swollen brain, intraperitoneally or intragastrically, a rapid attenuation of brain swelling was observed (Gojkovic et al., 2021a). A similar syndrome also appeared with peripherally induced syndromes, i.e., an occluded superior mesenteric artery (Knezevic et al., 2021a) or vein (Knezevic et al., 2021b), or both artery and vein (Knezevic et al., 2021a). Commonly, as in the present study, BPC 157 therapy rapidly eliminated the increased pressure in the superior sagittal sinus, severe portal and vena caval hypertension, and aortal hypotension and moreover quickly recruited collateral vessels, which abrogated venous and arterial thrombosis (Gojkovic et al., 2021a; Knezevic et al., 2021a; Knezevic et al., 2021a; Gojkovic et al., 2021b; Knezevic et al., 2021b; Strbe et al., 2021). This was interpreted as a widespread resolution of the Virchow triad (endothelium injury, hypercoagulability, and stasis), which allowed recovery from organ lesions (Vukojevic et al., 2018; Gojkovic et al., 2020; Kolovrat et al., 2020; Gojkovic et al., 2021a; Knezevic et al., 2021a; Knezevic et al., 2021a; Gojkovic et al., 2021b; Knezevic et al., 2021b; Strbe et al., 2021). Evidently, in the resolution of damage due to increased intra-abdominal hypertension, peripherally (Vukojevic et al., 2018; Gojkovic et al., 2020; Kolovrat et al., 2020; Knezevic et al., 2021a; Knezevic et al., 2021a; Knezevic et al., 2021b), centrally (Gojkovic et al., 2021a), or both peripherally and centrally (Gojkovic et al., 2021b; Strbe et al., 2021), there is a common therapeutic point from which BPC 157 operates.

Moreover, as BPC 157 therapy also works in advance, the properly reactivated azygos vein pathway and improved functioning of the combined inferior caval vein and left superior caval vein may resist even higher intra-abdominal hypertension (25 mmHg˂30 mmHg˂40 mmHg˂50 mmHg) and prolonged intra-abdominal pressures increases (25–120 min). There were no lethal outcomes despite the permanent maintenance of high intra-abdominal pressures (note that abdominal compartment syndrome with a sustained level of 25 mmHg may be fatal within 1 h (Strang et al., 2020)). As an accurate conceptual analogy with the similar therapeutic effect in occlusion syndromes (Vukojevic et al., 2018; Gojkovic et al., 2020; Kolovrat et al., 2020; Gojkovic et al., 2021a; Knezevic et al., 2021a; Knezevic et al., 2021a; Knezevic et al., 2021b) or alcohol and lithium intoxication (Gojkovic et al., 2021b; Strbe et al., 2021), BPC 157 therapy is effective against severe bradycardia and ST-elevation until asystole, myocardial congestion, and infarction before death. This beneficial effect meant that, with more severe intra-abdominal hypertension, BPC 157 rats still exhibited normal microscopic presentation of the heart. Thus, as before (Vukojevic et al., 2018; Gojkovic et al., 2020; Kolovrat et al., 2020; Gojkovic et al., 2021a; Knezevic et al., 2021a; Knezevic et al., 2021a; Gojkovic et al., 2021b; Knezevic et al., 2021b; Strbe et al., 2021), this activated alternative blood flow was provided continuously maintained heart function, leading to near-normal lung, liver, and kidney presentation, unlike the extreme congestion and hemorrhage observed in control rats. Collectively, these findings implicate that the heart, lungs, liver, and kidney are BPC 157 therapeutic targets.

Thus, despite increased intra-abdominal pressure, BPC 157 therapy normalized portal and caval pressure and aortal pressure, as well as portal vein and inferior caval vein and aorta presentation. This maintenance may be essentially important. Otherwise, high portal and caval hypertension, aortal hypotension, exaggerated congestion of both the inferior caval and superior mesenteric veins, and a narrowed aorta all appear along with the most severe organ lesions. This clear damage has also been seen in other vessel occlusion studies (Vukojevic et al., 2018; Gojkovic et al., 2020; Kolovrat et al., 2020; Gojkovic et al., 2021a; Knezevic et al., 2021a; Knezevic et al., 2021a; Gojkovic et al., 2021b; Knezevic et al., 2021b; Strbe et al., 2021). Conceptually, the gastrointestinal, liver, and kidney lesions described here are illustrative cause-consequence relationships indicative of an uninterrupted injurious course. Vice versa, when the lesions are absent/abrogated, they clearly illustrate the therapeutic effect of BPC 157 and an interrupted injurious course.

Thus, specific conceptual support in rats with high intra-abdominal pressures is provided by gastrointestinal tract failure, hemorrhagic lesions in the stomach, transmural hyperemia of the entire gastrointestinal tract, stomach, duodenum, and small and large bowel wall. The reduction of villi in the intestinal mucosa and crypt reduction with focal denudation of superficial epithelia and dilatation of the large bowel illustrate vascular failure (Chan et al., 2014). Accordingly, the liver and the kidney exhibited huge vascular congestion. Vice versa, the normalized portal and caval pressure and aortal pressure as a cause-consequence are convincing evidence of the functioning “bypassing key” (i.e., the azygos vein). Consequently, BPC 157-treated rats exhibited no or minimal congestion in the gastrointestinal mucosa, with well-preserved intestinal villi and colonic crypts and no dilatation of the large bowel, as well as a maintained vascular supply and reduced vascular failure (Chan et al., 2014). In the liver and kidney, only mild congestion was observed at the highest intra-abdominal pressures.

Furthermore, high intra-abdominal pressures/increased intracranial pressures led to the severe presentation of brain lesions. Equally, with therapy, the reversed injury course (increased intra-abdominal pressure/reduced intracranial hypertension) led to reduced intracranial hypertension as the ultimate therapeutic outcome when the venous system was supported (i.e., activation of the azygos shunt). This was key in the brain as well, as pressures were not rapidly transmitted up through the venous system, and thereby brain presentation was preserved. The brain was preserved both grossly (absent brain swelling) and microscopically (consistent beneficial effect in all brain areas). Evidently, the beneficial effect of BPC 157 acted against the full range of brain lesions, in the order cerebellum cortex > hypothalamus/thalamus > cerebral cortex. The cerebellar cortex appeared to be the most affected, and the cerebral cortex was the least affected. The hippocampus, with increased lesion severity at higher intra-abdominal pressures, may be seen as a particular target. On the other hand, the vicious course induced by high intra-abdominal pressure can be simultaneously initiated and perpetuated from different sites (it should be noted that intracranial hypertension may essentially cause pulmonary edema and impair pulmonary circulation (Chen, 2009)).

Both BPC 157 regimens (µg and ng) had a similar therapeutic effect in all of the investigated protocols of abdominal compartment syndrome. Further cause-consequence evidence could be seen in BPC 157-treated rats with high intra-abdominal pressures, as treatment largely abrogated both arterial and venous thrombosis. This was seen before with vessel occlusion (Vukojevic et al., 2018; Gojkovic et al., 2020; Kolovrat et al., 2020; Gojkovic et al., 2021a; Knezevic et al., 2021a; Knezevic et al., 2021a; Knezevic et al., 2021b), alcohol and lithium intoxication (Gojkovic et al., 2021b; Strbe et al., 2021), and abdominal aorta anastomosis (Hrelec et al., 2009). The effect occurred peripherally (i.e., the largest thrombosis initially (i.e., 25 mmHg) appeared just in the hepatic veins, resembling the presentation of Budd–Chiari syndrome (Gojkovic et al., 2020)), and centrally (superior sagittal sinus). Abrogated thrombosis, both peripherally and centrally (Vukojevic et al., 2018; Gojkovic et al., 2020; Kolovrat et al., 2020; Gojkovic et al., 2021a; Knezevic et al., 2021a; Knezevic et al., 2021a; Gojkovic et al., 2021b; Knezevic et al., 2021b), means that stasis was evidently avoided, or at least markedly reduced. Along with the “bypassing key” and rapidly activated collaterals, Virchow’s triad was consistently reduced, both peripherally and centrally (Vukojevic et al., 2018; Gojkovic et al., 2020; Kolovrat et al., 2020; Gojkovic et al., 2021a; Knezevic et al., 2021a; Knezevic et al., 2021a; Gojkovic et al., 2021b; Knezevic et al., 2021b; Strbe et al., 2021). In particular, BPC 157-induced endothelial maintenance (Sikiric et al., 1994) and the “bypassing key” (Vukojevic et al., 2018; Gojkovic et al., 2020; Kolovrat et al., 2020; Gojkovic et al., 2021a; Knezevic et al., 2021a; Knezevic et al., 2021a; Gojkovic et al., 2021b; Knezevic et al., 2021b; Strbe et al., 2021) occur along with the previously noted BPC 157-NO system interactions. This can involve the release of NO on its own (Sikiric et al., 1997; Turkovic et al., 2004), as well as maintained NO system function against NOS blockade (L-NAME) or overfunction (L-arginine) (for review, see Sikiric et al., 2014). Furthermore, blood pressure maintenance (Sikiric et al., 1997), maintained thrombocyte function (Stupnisek et al., 2015; Konosic et al., 2019), and vasomotor tone occurred through BPC 157-specific activation of the Src-caveolin-1-eNOS pathway (Hsieh et al., 2020). Besides, the “bypassing key” also occurred with minor vessel occlusion, showing a therapeutic effect. The “bypassing pathway” may be the inferior anterior pancreaticoduodenal vein (with a reduction in duodenal congestion lesions) (Amic et al., 2018) and arcade vessels (with a reduction in left colic vein and artery occlusion-induced ischemic reperfusion colitis) (Duzel et al., 2017). An effect was also seen with parietal peritoneum removal (fewer adhesions) (Cesar et al., 2020); in cecum perforation (after perforation (Drmic et al., 2018), unlike empty vessels (not visible), blood vessels were filled with blood and were thereby clearly presented as blood vessels running toward the defect, with less bleeding and increased healing); in bile duct ligation-induced liver cirrhosis (prevention and reversal of portal hypertension) (Sever et al., 2019). Likewise, given during reperfusion after clamping the common carotid arteries, BPC 157 reduced stroke (i.e., both early and delayed hippocampal neural damage, achieving full functional recovery in the Morris water maze test, inclined beam-walking test, and lateral push test) (Vukojevic et al., 2020) or reduced L-NAME-induced retinal ischemia in rats (Zlatar et al., 2021).

Furthermore, the adequate activation of alternative pathways should occur along with the additional (direct) beneficial effects on affected targets. In addition to venous occlusion-induced lesions (Vukojevic et al., 2018; Gojkovic et al., 2020; Kolovrat et al., 2020), BPC 157 is known to reduce lesions in the entire gastrointestinal tract (Sikiric et al., 1994; Ilic et al., 2009; Sever et al., 2009; Ilic et al., 2010; Ilic et al., 2011a; Ilic et al., 2011b; Petrovic et al., 2011; Lojo et al., 2016; Drmic et al., 2017; Becejac et al., 2018). Likewise, BPC 157 may reduce lesions in the liver (Sikiric et al., 1993b; Ilic et al., 2009; Ilic et al., 2010; Ilic et al., 2011a; Ilic et al., 2011b; Lojo et al., 2016; Drmic et al., 2017), including liver cirrhosis, induced by bile duct ligation (Sever et al., 2019) or continuous alcohol consumption (Prkacin et al., 2001). Also, BPC 157 may prevent and reverse chronic heart failure induced by doxorubicin application (Lovric-Bencic et al., 2004). BPC 157 reduces various arrhythmias (i.e., potassium overdose-induced hyperkalemia (Barisic et al., 2013), digitalis (Balenovic et al., 2009), neuroleptics (i.e., prolonged QTc-intervals that may also be centrally related) (Strinic et al., 2017), bupivacaine (Zivanovic-Posilovic et al., 2016), lidocaine (Lozic et al., 2020), and succinylcholine (Stambolija et al., 2016)). Likewise, BPC 157 reduces lung congestion after vessel occlusion (Vukojevic et al., 2018; Gojkovic et al., 2020; Kolovrat et al., 2020; Gojkovic et al., 2021a; Knezevic et al., 2021a; Knezevic et al., 2021a; Gojkovic et al., 2021b; Knezevic et al., 2021b; Strbe et al., 2021), intratracheal alcohol instillation (Stancic-Rokotov et al., 2001a; Stancic-Rokotov et al., 2001b), and pulmonary hypertension syndrome in chickens (Grabarevic et al., 1997) and in monocrotaline-treated rats (Udovicic et al., 2021). As a recently reviewed subject (Vukojevic et al., 2022), BPC 157 has been shown to reduce brain lesions, trauma-induced brain injury (Tudor et al., 2010), compression-induced spinal cord injury (Perovic et al., 2019), and stroke (Vukojevic et al., 2020). In addition, BPC 157 reduces severe encephalopathies (NSAID overdose, Ilic et al., 2010; Ilic et al., 2011a; Ilic et al., 2011b; Lojo et al., 2016; Drmic et al., 2017), neurotoxin cuprizone-induced multiple sclerosis in a rat model (Klicek et al., 2013), and magnesium overdose (Medvidovic-Grubisic et al., 2017)). Importantly, BPC 157 also reduces the consequences of, i.e., gastrointestinal and/or liver lesions (Ilic et al., 2010; Ilic et al., 2011a; Ilic et al., 2011b; Lojo et al., 2016; Drmic et al., 2017) and severe muscle weakness (Klicek et al., 2013; Medvidovic-Grubisic et al., 2017)). Thus, these beneficial effects are interrelated and appear useful for the therapy of multiple vicious circles that may simultaneously appear in rats permanently maintained under severe intra-abdominal hypertension conditions. By themselves, all these disturbances, which were ameliorated/reduced, are quite severe. Considering the different causes of secondary abdominal compartment syndrome (Hunter and Damani, 2004; Hedenstierna and Larsson, 2012), these disturbances, each with a different set of causes, may also contribute to high intra-abdominal pressure, and thus when ameliorated/reduced, they may indicate the beneficial effect of BPC 157 therapy in cases of secondary high intra-abdominal pressure. There, due to its beneficial effect on damaged muscle and the recovery of its function (Staresinic et al., 2006; Novinscak et al., 2008; Mihovil et al., 2009; Pevec et al., 2010; Kang et al., 2018), it is possible that the BPC 157 therapeutic effect may also be related to improvements in abdominal wall compliance.

Finally, a summary would reveal a consistent demonstration of particular beneficial effects, i.e., the activation of collateral pathways related to the injurious occlusion (Vukojevic et al., 2018; Gojkovic et al., 2020; Kolovrat et al., 2020; Gojkovic et al., 2021a; Knezevic et al., 2021a; Knezevic et al., 2021a; Gojkovic et al., 2021b; Knezevic et al., 2021b; Strbe et al., 2021), with one or two vessel ligations (Vukojevic et al., 2018; Gojkovic et al., 2020; Kolovrat et al., 2020; Gojkovic et al., 2021a; Knezevic et al., 2021a; Knezevic et al., 2021a; Knezevic et al., 2021b) or more (high intra-abdominal hypertension compressing all blood vessels), with either specific injury (vessel ligation) (Vukojevic et al., 2018; Gojkovic et al., 2020; Kolovrat et al., 2020; Gojkovic et al., 2021a; Knezevic et al., 2021a; Knezevic et al., 2021a; Knezevic et al., 2021b) or broad non-specific injuries (alcohol and lithium intoxication (Gojkovic et al., 2021b; Strbe et al., 2021) and intra-abdominal hypertension (present study)). These particular beneficial effects, i.e., the activation of collateral pathways, should occur along with the activated molecular pathways (Tkalčević et al., 2007; Chang et al., 2011, 2014; Huang et al., 2015; Hsieh et al., 2017; Kang et al., 2018; Vukojevic et al., 2018; Wang et al., 2019; Cesarec et al., 2013; Hsieh et al., 2020; Park et al., 2020; Vukojevic et al., 2020; Wu et al., 2020), illustrative of the complexity of the processes involved. Not only in theory but these results should also be combined with extensive studies on how BPC 157 exerts its specific effects. In one study, it affected *Egr*, *Nos*, *Srf*, *Vegfr*, *Akt1*, *Plcɣ,* and *Kras* gene expression in the vessel that provides an alternative operating pathway (i.e., the left ovarian vein as the key for infrarenal occlusion-induced inferior vena cava syndrome in rats) (Vukojevic et al., 2018). In the hippocampus, BPC 157 strongly elevates *Egr1*, *Akt1*, *Kras*, *Src*, *Foxo*, *Srf*, *Vegfr2*, *Nos3,* and *Nos1* expression and decreases *Nos2* and *Nfkb* expression; these changes may indicate how BPC 157 exerts its effects (Vukojevic et al., 2020). Additionally, mitigated leaky gut syndrome suggests that BPC 157 is a stabilizer of cellular junctions by increasing tight junction protein ZO-1 expression and transepithelial resistance (Park et al., 2020). A reduction in the mRNA level of inflammatory mediators (iNOS, IL-6, IFN-γ, and TNF-α) and increased expression of HSP 70 and 90 and antioxidant proteins such as HO-1, NQO-1, glutathione reductase, glutathione peroxidase 2, and GST-pi were observed (Park et al., 2020). These findings clearly show that BPC 157 may successfully compete with the initial events in intra-abdominal hypertension (i.e., significant damage to the intestinal epithelium and dilation of intestinal tight junctions, increased mucosal barrier permeability, bacterial translocation, and sepsis (Gong et al., 2009)). Of note, the antioxidant effects of BPC 157 (Belosic Halle et al., 2017; Luetic et al., 2017; Sucic et al., 2019) occurred in both ischemic and reperfusion conditions in various tissues (i.e., colon, duodenum, cecum, liver, and veins) and plasma, in vessel occlusion studies in particular (Vukojevic et al., 2018; Kolovrat et al., 2020; Knezevic et al., 2021a; Knezevic et al., 2021a; Knezevic et al., 2021b).

Coming back to the mentioned general theoretic cytoprotection effects (Robert, 1979; Szabo et al., 1985; Sikiric et al., 2010; Sikiric et al., 2018), it should be noted that Robert’s cytoprotection generally holds a defensive response against direct injuries. Possibly, as a final clue supporting cytoprotection theory, this upgraded defensive principle (i.e., preserved endothelial function promotes the organization of additional bypassing collaterals functioning to compensate against the ongoing injurious course; for review, see Sikiric et al., 2018) persists against the notion of continuous, direct injury (increased intra-abdominal hypertension) and shows that organs may adequately function despite continuously elevated intra-abdominal pressure.

Finally, calvariectomy and/or laparotomy, used in therapy to reduce abdominal compartment syndrome (Hunter and Damani, 2004; Hedenstierna and Larsson, 2012), and in the present study to assess intracranial (superior sagittal sinus), portal, inferior caval vein, and aortal pressure, and brain, organ, and vessel presentation, may not interfere with the worst circumstances created in the abdominal compartment syndrome. In fact, the evidence shows that superior sagittal sinus hypertension even increased slightly after laparotomy. Thereby, the evidenced severe superior sagittal sinus, portal, and caval hypertension and aortal hypotension occurred along with the rapid worsening that would appear along with decompression (Hsu et al., 2004). The reduction with BPC 157 is along with its previous reducing potential on severe superior sagittal sinus, portal, and caval hypertension and aortal hypotension (Vukojevic et al., 2018; Gojkovic et al., 2020; Kolovrat et al., 2020; Gojkovic et al., 2021a; Knezevic et al., 2021a; Knezevic et al., 2021a; Gojkovic et al., 2021b; Knezevic et al., 2021b; Strbe et al., 2021).

In conclusion, these findings related to BPC 157 therapy may be important in both shorter and more prolonged periods of abdominal compartment syndrome development and reduction. Of note, intra-abdominal hypertension is quite frequent in critically ill patients and the cause of multiorgan dysfunction (Hunter and Damani, 2004; Hedenstierna and Larsson, 2012). Also, we should acknowledge that animal models although quite different (Schachtrupp et al., 2007) (here, 25, 30, 40, and 50 mm Hg by intraperitoneal insufflation of ordinary air controlled and maintained by a manual manometer leads to invariable abdominal compartment syndrome), correlate fairly well with the circumstances in humans. Therefore, in principle, the application of pentadecapeptide BPC 157 therapy is effective in particular venous occlusion syndromes, as well as for recovery from all compressed blood vessels and the consequent syndrome (Vukojevic et al., 2018; Gojkovic et al., 2020; Kolovrat et al., 2020; Gojkovic et al., 2021a; Knezevic et al., 2021a; Knezevic et al., 2021a; Gojkovic et al., 2021b; Knezevic et al., 2021b; Strbe et al., 2021). Fully achieved reduction of severe lesions in the brain, heart, lungs, liver, kidneys, and gastrointestinal tract reduced thrombosis in both veins and arteries, peripherally and centrally, and fully abrogated intracranial (superior sagittal sinus), portal, and caval hypertension and aortal hypotension may be regarded as a proof of concept. This study provides evidence of reductions in all the consequences of intra-abdominal hypertension, even grade III and grade IV, which may not be concerned by the relative paucity of BPC 157 clinical data (Sikiric et al., 2018; Seiwerth et al., 2021; Vukojevic et al., 2022). BPC 157 has also been shown to be efficacious in ulcerative colitis (for review, see Sikiric et al., 2011; Sikiric et al., 2012; Sikiric et al., 2013; Sikiric et al., 2018; Sikiric et al., 2020b), in both the clinical setting (Veljaca et al., 2003; Ruenzi et al., 2005) and the experimental animal models (for review, see Sikiric et al., 2011; Sikiric et al., 2012; Sikiric et al., 2013; Sikiric et al., 2018; Sikiric et al., 2020b) and complications (for review, see Sikiric et al., 2020b). An important point regarding application in practice includes various species (i.e., Tlak Gajger et al., 2018). However, the most important advantage is the very safe profile of BPC (the lethal dose (LD1) could be not achieved) (Seiwerth et al., 2018), emphasized in terms of its physiological role (assessed using *in situ* hybridization and immunostaining for BPC 157 in the human gastrointestinal mucosa, lung bronchial epithelium, the epidermal layer of the skin, and kidney glomeruli) (Seiwerth et al., 2018). This point was recently confirmed in a large study by Xu and collaborators (Xu et al., 2020). In this context, also for practical purposes, providing that the therapeutic effects speak for themselves, we provide a good background for further application of BPC 157 as a therapy.

## Data Availability

The raw data supporting the conclusion of this article will be made available by the authors, without undue reservation.
