# Corrigendum: Stable Gastric Pentadecapeptide BPC 157 Therapy for Primary Abdominal Compartment Syndrome in Rats

**DOI:** 10.3389/fphar.2021.844785

**Published:** 2022-01-21

**Authors:** Marijan Tepes, Slaven Gojkovic, Ivan Krezic, Helena Zizek, Hrvoje Vranes, Zrinko Madzar, Goran Santak, Lovorka Batelja, Marija Milavic, Suncana Sikiric, Ivica Kocman, Karol Simonji, Mariam Samara, Mario Knezevic, Ivan Barisic, Eva Lovric, Sanja Strbe, Antonio Kokot, Ivica Sjekavica, Toni Kolak, Anita Skrtic, Sven Seiwerth, Alenka Boban Blagaic, Predrag Sikiric

**Affiliations:** ^1^ Department of Surgery, General Hospital Nasice, Nasice, Croatia; ^2^ Department of Clinical Medicine, Faculty of Dental Medicine and Health Osijek, Osijek, Croatia; ^3^ PhD Program Translational Research in Biomedicine—TRIBE, School of Medicine, University of Split, Split, Croatia; ^4^ Department of Pharmacology, School of Medicine, University of Zagreb, Zagreb, Croatia; ^5^ Clinical Department of Surgery, Sestre Milosrdnice University Hospital Center, Zagreb, Croatia; ^6^ Department of Surgery, Faculty of Medicine, University of Osijek, Osijek, Croatia; ^7^ Department of Pathology, School of Medicine, University of Zagreb, Zagreb, Croatia; ^8^ Internal Diseases Clinic, Faculty of Veterinary Medicine Zagreb, Zagreb, Croatia; ^9^ Department of Anatomy and Neuroscience, Faculty of Medicine, J.J. Strossmayer University of Osijek, Osijek, Croatia; ^10^ Department of Diagnostic and Interventional Radiology, University Hospital Centre, Zagreb, Croatia; ^11^ Department of Surgery, School of Medicine, University of Zagreb, Zagreb, Croatia

**Keywords:** gastric pentadecapeptide BPC 157, primary abdominal compartment syndrome, rats, brain edema, lung edema

In the published article, there was an error in **affiliations 2** and **3**. Instead of “^2^Department of Clinical Medicine, Faculty of Dental Medicine and Health Osijek, Zagreb, Croatia; ^3^PhD Program Translational Research in Biomedicine—TRIBE, School of Medicine, University of Split, Zagreb, Croatia.,” it should be “^2^Department of Clinical Medicine, Faculty of Dental Medicine and Health Osijek, Osijek, Croatia; ^3^PhD Program Translational Research in Biomedicine—TRIBE, School of Medicine, University of Split, Split, Croatia.”

The authors apologize for this error and state that this does not change the scientific conclusions of the article in any way. The original article has been updated.

