# Supplementary figures and images for: Stable Gastric Pentadecapeptide BPC 157 Therapy for Primary Abdominal Compartment Syndrome in Rats
Source: Front Pharmacol. 2021 Dec 13;12:718147. doi: 10.3389/fphar.2021.718147 (PMC8710746; doi:10.3389/fphar.2021.718147)

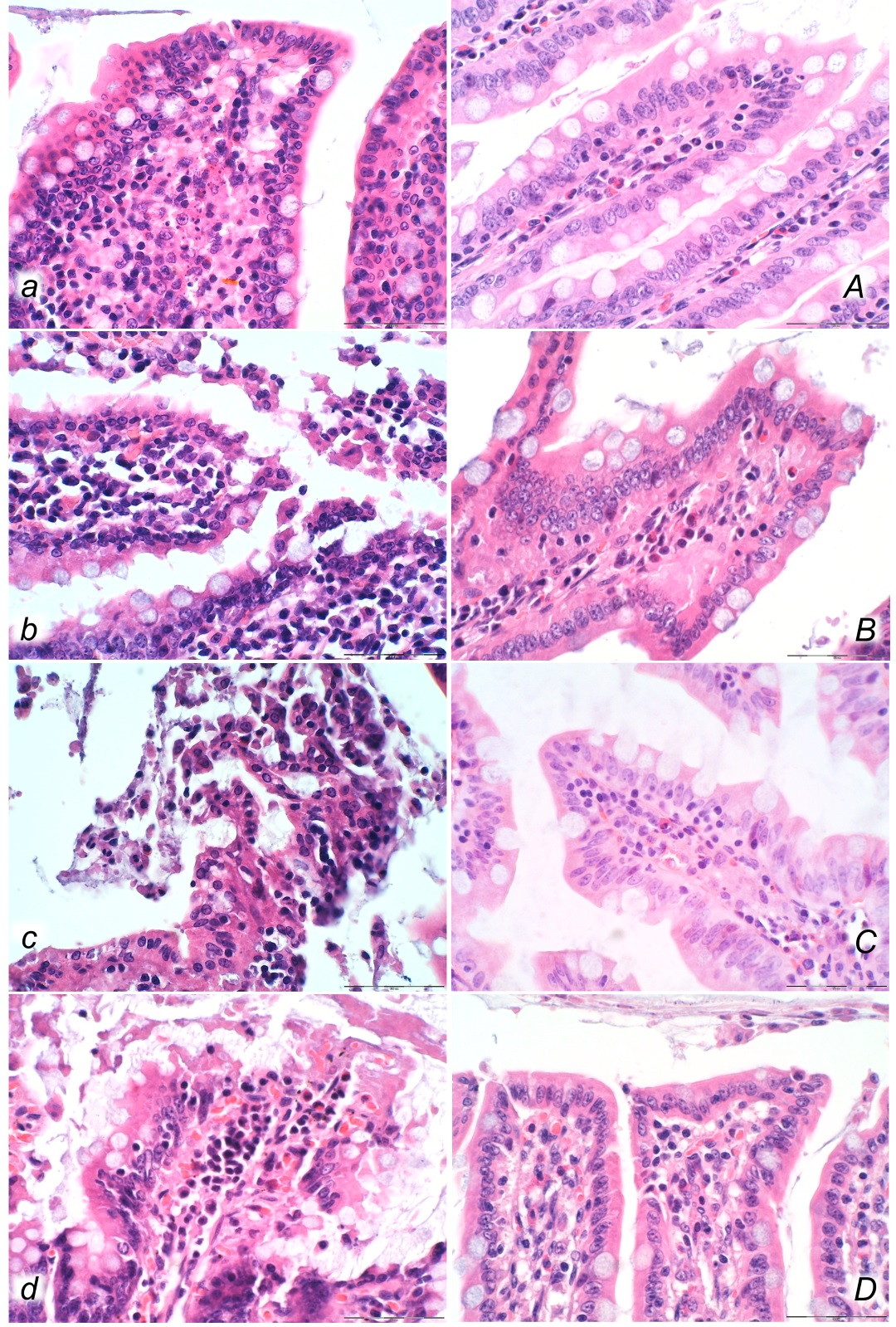

Supplement: Supplementary file 1 [file Image2.TIF]

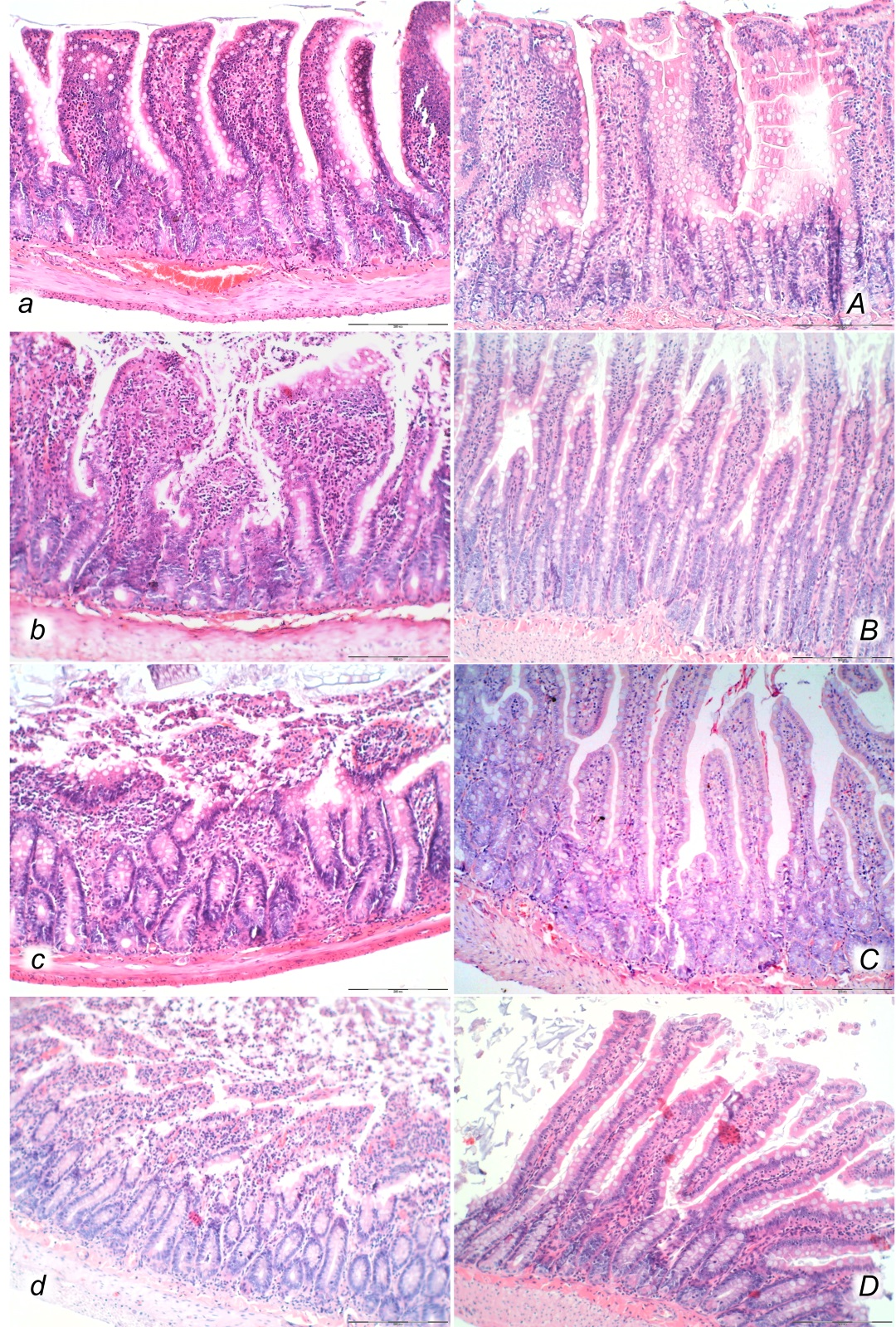

Supplement: Supplementary file 2 [file Image1.TIF]
